# Molecular mechanisms of miR-192 in cancer: a biomarker and therapeutic target

**DOI:** 10.1186/s12935-025-03666-5

**Published:** 2025-03-14

**Authors:** Yang Yang, Siti Razila Abdul Razak, Ida Shazrina Ismail, Yanxia Ma, Muhammad Amir Yunus

**Affiliations:** 1 Department of Biomedical Sciences, Advanced Medical and Dental Institute, Universiti Sains Malaysia, Bertam, Kepala Batas, Pulau Pinang, Malaysia; 2 School of Medical Technology, Shaanxi University of Chinese Medicine, Xianyang, Shaanxi China

**Keywords:** miR-192, Expression, Tumor suppressor, Oncogene, Mechanism, Biomarkers

## Abstract

Cancer remains a major global health challenge due to its rising prevalence and high mortality rates. The field of microRNAs (miRNAs) has made significant progress in the understanding of tumorigenesis and has broadened our knowledge of their targeting, especially in cancer therapy. miRNAs, a class of small non-coding RNAs, participate in post-transcriptional gene regulation by translational inhibition or mRNA degradation. Among these, microRNA-192 (miR-192) is located on human chromosome 11q13.1, and is highly correlated with the occurrence and development of various human cancers. Dysregulation of miR-192 has been extensively studied in various pathological processes, including tumorigenesis, making it a valuable biomarker for cancer diagnosis and prognosis. The functional role of miR-192 varies across cancer types, acting as either a tumor suppressor or as an oncogene through the modulation of multiple gene expressions and downstream signaling pathways. However, the roles of miR-192 in cancer appear inconsistent across types, with current research often focused on specific genes or pathways, limiting insight into its broader impact on cellular signaling networks. Therefore, this review aims to provide a comprehensive overview of miR-192 research. The paper reviews differences in miR-192 expression in cancer and systematically summarizes the role of miR-192 in cancers. The review further explores the complex roles of miR-192 in various pathological processes, emphasizing its regulatory pathways, interaction networks, and association with tumor progression. This review also illustrates the clinical application of miR-192 as a diagnostic and prognostic biomarker for non-invasive cancer detection, as it is consistently present in both serum and exosomes. A comprehensive summary and analysis of the relationship between miR-192 and various cancers may provide valuable insights, potentially guiding novel approaches in clinical diagnosis, therapeutic strategies, and foundational cancer research.

## Background

Cancer poses a significant global public health challenge due to its increasing prevalence and high mortality rate. There remains an urgent need to identify novel diagnostic markers and therapeutic targets to improve patient outcomes [1, 2]. The exploration of molecular targets, particularly in the field of non-coding RNAs, has highlighted miRNAs as promising candidates due to their extensive regulatory roles in various pathological processes, including tumorigenesis [3]. Essentially, miRNAs are small non-coding RNA molecules (around 22 nucleotides) that regulate gene expression at the post-transcriptional level in a wide range of biological contexts [4, 5].

Furthermore, miRNAs are distinguished by a “seed” sequence, typically 7–8 nucleotides long, which binds to the 3'-untranslated regions (3'-UTRs) of target mRNAs, influencing mRNA stability and translation. The human miR-192 sequence, specifically the 24-CUGACCUAUGAAUUGACAGCC-44 seed (https://www.mirbase.org/search.shtml), is located on the human chromosome 11q13.1 [6]. miRNA biogenesis is a multi-step process involving transcription of primary miRNAs (pri-miRNAs) by RNA polymerase II or III, processed by the Drosha-DiGeorge Syndrome Critical Region 8 (Drosha-DGCR8) complex into precursor miRNAs (pre-miRNAs), with further maturation in the cytoplasm by Dicer to form the mature miRNA duplex [7–11]. For miR-192, the guide strand (miR-192-5p, originating from the 5’ side of the pre-miRNA) binds to the argonaut protein (AGO) to form the miRNA-induced silencing complex (RISC). Meanwhile, the passenger strand (miR-192-3p, the complementary 3’ strand) is usually degraded. Both miR-192-3p and miR-192-5p, however, target genes implicated in oncogenic processes, affecting cell proliferation, migration, invasion, epithelial-mesenchymal transition (EMT), angiogenesis, and drug resistance [12]. Both miR-192-3p and miR-192-5p target specific genes involved in cancer, thereby influencing key cellular processes, including cell proliferation, migration, invasion, EMT, angiogenesis and drug resistance.

miR-192 is aberrantly expressed across multiple cancer types, where it exerts either oncogenic or oncostatic effects, making it a promising target in cancer therapy. This dual role indicates its complex and context-dependent impact on cancer biology. Despite the growing evidence of miR-192's involvement in cancer, its functional roles appear to vary across different cancer types, and the consistency of these roles remains uncertain. Current research focuses on isolated target genes or specific signaling pathways, which limits our understanding of miR-192’s broader influence within the intricate network of cellular signaling pathways involved in cancer progression.

In understanding miR-192’s role in cancer, this review consolidates current insights into the aberrant expression, multifaceted functions, and complex regulatory mechanisms of miR-192 across various cancers. By highlighting its upstream regulators, downstream targets, and associated signaling pathways, The review aims to provide a comprehensive understanding of miR-192’s role in cancer progression. Additionally, its potential clinical applications as a diagnostic and prognostic biomarker are highlighted.

## Expression of miR-192 in various cancers

miR-192 exhibits dynamic expression patterns that vary across tissues. It is highly expressed in certain normal tissues, such as the liver, kidney, intestine, and epithelial tissues, while its expression may be minimal in others. Notably, miR-192 expression is often dysregulated in cancers, where it undergoes significant alterations. A summary of miR-192 expression patterns in different cancers is provided in Table 1.

**Table 1:** The expression of miR-192 in cancers

Refs	Disease	miRNA	Expression	Samples	Cell lines
[12]	Cholangiocarcinoma (CCA)	miR-192-5p	Up	21 CCA tissues vs. 21 ANT	CCA cells (TFK-1, CCLP-1, HCCC 9810, RBE, HuCCT-1) vs. HIBECs (Human intrahepatic biliary epithelial cells)
[13]	CCA	miR-192	Up	94 CCA patients undergoing tumor resection and 40 HCs	
[14]	CCA	miR-192	Up	Urinary samples from 22 CCA patients vs. 21 HCs	
[15]	CCA	miR-192	Up	Serum from 11 CCA patients vs. 9 HCs	
[16]	Cervical cancer	miR-192-5p	Up	20 adenocarcinoma tissues vs. 20 normal tissues	
[17]	HNSCC	miR-192	Up	Hypoxic sEVs vs. normoxic sEVs	
[18]	Nasopharyngeal carcinoma (NPC)	miR-192	Up	76 NPC tissues vs. 76 normal tissues	NPC cells C666-1 vs. human immortalized nasopharyngeal epithelial cell line NP69
[19]	Breast cancer (BC)	miR-192	Down	58 BC tissues vs. 58 ANT	BC cells MCF-7 and MDA-MB-231 vs. NCs Hs578Bst
[20]	BC	miR-192	Down	BC tissues (15 ER/PR-positive samples and 15 ER/PR-negative samples) vs. ANT	
[21]	BC	miR-192-5p	Down		DOX-resistant BC cells MCF-7/ADR vs. MCF-7 vs. NCs MCF-10A
[91]	TNBC	miRNA-192	Up		TNBC cells HCC38, HCC1395, and HCC1937 treated with vs. without trichostatin A
[22]	Glioma	miR-192-5p	Down	50 glioma tissues vs. ANT	Glioma cells (A172, U87, LN18, U251, LN229, and U118) vs. normal human astrocyte cells (NHA)
[23]	Glioblastoma multiforme (GBM)	MiR-192-5p	Down		GBM cells (U251, A172, U87, T98G, and GL15) vs. human astrocyte (HA)
[24]	Medulloblastoma	miR-192	Down	29 medulloblastoma tissues vs. the normal cerebellum;	Medulloblastoma cells (D283, MED8A and UW228) vs. normal cerebellum
[48]	Esophageal cancer (EC)	miR-192-5p	Up	The plasma of 44 EC patients without neoadjuvant chemotherapy vs. 55 HCs	
[49]	EC	miR-192	Up	50 primary ESCC tissues vs. 50 ANT	EC cells KYSE-150, KYSE-510, EC-9706 vs. immortalized human esophageal epithelial cell
[50]	EC	miR-192	Down	80 post-therapeutic EC tissues vs. corresponding pretherapeutic tissues	
[42]	Colorectal cancer (CRC)	MiR-192-5p	Down	The serum of 164 colon cancer patients vs. 60 HCs	
[25]	CRC	miR-192-5p	Down		SW480 and HCT116 vs. human normal colonic epithelial cells
[26]	CRC	miR-192	Down		HCT-116、HT-29、SW480、RKO vs FHC
[27]	CRC	miR-192-5p	Down	Tumor tissues from 8 patients with liver metastasis after radical operation vs. 8 patients without liver metastasis after radical operation (all patients confirmed colon cancer stage IIIB)	
[28]	CRC	miR-192	Down	49 colon cancer tissues vs. 49 ANT	
[29]	CRC	miR-192	Down	Specimens from 29 individual colon cancer patients at stages I (n = 9), II (n = 8) or IV(n = 12) vs. HCs	Metastatic HCT116, RKO, RCA and GEO cells vs. non-metastatic or low-metastatic FET and HCT116b cells
[30]	CRC	miR-192-5p	Down	30 CRC tumors tissues vs. 30 ANT	CRC cells sw480 and LOVO vs. the normal colon epithelial cell line FHC
[31]	CRC	miR-192	Down	107 CRC tissues vs. 107 ANT	CRC cells (HT-29, HCT-116 and SW-620) vs. 3 NATs
[46]	Bladder cancer	miR-192	Down	Morning urine from 118 bladder cancer vs. 120 benign urinary system diseases (control)	
[32]	Bladder cancer	miR-192-5p	Down	Human bladder cancer tissues vs. control	Human bladder cancer cell lines vs. control
[41]	Endometrial cancer	miR-192-5p	Down	TAMs-derived exosomes vs. control	
[40]	Endometrial cancer	miR-192-5p	Down	56 endometrial carcinoma tissues vs. 56 ANT	
[39]	Nephroblastoma	miR-192	Down	All nephroblastoma subtypes tissues (4 epithelial-type, 6 blastema-type, 11 mixed-type, 3 stroma-type, and 9 nephrogenic rests) vs. 7 mature kidney parenchyma	
[44]	Pediatric AML	miR-192	Down	Serum from 97 AML cases vs. 50 HCs	
[45]	AML	miR-192	Down	Specimens from AML patients vs. HCs	
[47]	CLL	miR-192	Down	PBMCs of 20 CLL patients vs. 20 HCs	
[33]	PTC	miR-192-5p	Down	20 PTC species vs. 20 ANT	PTC cells TPC-1、BHT101 and B-CPAP vs Human thyroid follicular epithelial cell line Nthy-ori 3–1
[34]	Osteosarcoma (OSA)	miR-192	Down	46 tumor tissues vs. ANT	OSA cells (MG-63, U2-OS, 143B and SOSP-9607) vs. normal osteoblasts (hFOB1.19 cells)
[35]	OSA	miR-192	Down	22 OSA tissues vs. 22 ANT	Human OSA cells MG63 vs. normal osteoblast hFOB1.19 cells
[36]	OSA	miR-192-5p	Down	25 OSA tissues vs. 25 ANT	OSA cells 143B and U2OS vs. hFOB (normal human osteoblast cell line)
[37]	OSA	miR-192	Down	20 OSA tissue vs. 20 ANT	OSA cell lines (U2OS and MG63) vs. human osteoblast cell line
[38]	OSA	miR-192	Down	80 OSA tissues vs. corresponding noncancerous bone tissues	
[52]	Ovarian carcinoma *	miR-192-5p	Down	Plasma exosomes from EOC patients vs. HCs	
[51]	Ovarian carcinoma *	miR-192	Up	3 mucinous carcinomas vs. 53 high-grade serous carcinomas or 17 endometrioid carcinomas or 7 low-grade serous carcinomas or 5 clear cell carcinomas or 4 borderline tumors	
[77, 78]	Lung cancer*	miR-192	Up		cisplatin-resistant cell line A549/DDP vs the lung cancer cisplatin-sensitive cell line A549
[72]	Lung cancer*	miR-192-5p	Down	Serum samples from 68 lung cancer patients with bone metastasis vs. 78 lung cancer patients without bone metastasis or 130 HCs	Human lung cancer cells (H1299、H1650、PC9、A549、) vs. normal lung epithelial cells (BEAS-2B)
[71]	Lung cancer*	miR-192	Down	17 lung cancer tissues vs. ANT	
[74]	Lung cancer*	miR-192	Up		A549 cells vs. normal human bronchial epithelial cells
[75]	Lung cancer*	miR-192-5p	Up		Lung cancer cells A549 vs. normal lung epithelial cells NCL-H460 and BEAS-2E
[76]	Lung cancer*	miR-192	Up		H520, H661, A549, H23, H1975, HCC827, PC9, H460 and Calu-6 cell lines vs. H1703
[73]	Lung cancer*	miR-192	Down	Bronchial lavage samples from NSCLC participants vs. 100 HCs	
[110]	Lung cancer*	miR-192-5p	Up		NSCLC cells H460 and A427 were treated with curcumin vs. with DMSO
[57]	Gastric cancer (GC)*	miR-192-5p	Up	The exosome in plasma samples from 60 GC patients vs. 63 HCs	
[53]	GC*	miR-192-5p	Up	30 GC tissues vs. ANT	
[54]	GC*	miR-192	Up	30 GC tissues vs. normal tissues	
[55]	GC*	miR-192	Up	GC tissues vs. ANT	
[56]	GC*	miR-192	Up	7 GC tissues vs. 3 ANT (microarray) 23 GC tissus vs. ANT (qRT-PCR)	GC cells (NCI-N87, KATO III, RF-48, AGS) vs. normal human gastric epithelial cell line (HFE145)
[58]	GC*	miR-192	Up	Plasma of 48 GC/DM (gastric cancer with distant metastasis) patients vs. 48 GC/NDM (gastric cancer with no distant metastasis) and 30 HCs	
[60]	GC*	miR-192-5p	Down		the cisplatin-resistant GC cells SGC7901/DDP vs. GC cells SGC7901
[43]	GC*	miR-192-5p	Down/no difference	25 GC tissues vs. ANT (down) Serum of 25 GC patients vs. HCs (no difference)	
[59]	GC*	miR-192	No difference/down	118 GC tissues vs. 118 ANT (no difference)	GC cells (MGC-803, BGC-823 and SGC-7901) vs. normal gastric tissues (randomly selected from previous 118 cases as controls)
[63]	Hepatocellular carcinoma (HCC)*	miR-192-5p	Down	HCC tissues vs. normal controls (TCGA)	
[61]	HCC*	miR-192	Down	50 HBV-related HCC tissues vs. 50 ANT	
[62]	HCC*	miR-192	Down	101 HCC primary tumors tissues vs. 101 ANT Tumor vs. normal (TCGA) Primary HCCs with vascular tumor cell invasion vs. those without (TCGA)	HCC-LM3 cells vs. MHCC-97L vs. MHCC-97L (with gradually decreasing metastatic potential)
[64]	HCC*	miR-192	Up	Liver tissue from HCC-rats treated with thymoquinone vs. HCC-rats with control	
[66]	HCC*	miR-192-5p	Up		HepG2 cells treated with vs. without Solamargine (SM)
[119]	HCC*	miR-192	Up	Exosomes from the plasmas of 24 HCC patients vs. 20 HCs (microarray) Exosomes from the plasmas of 84 HCC patients vs. 20 HCs (qRT-PCR)	
[67]	HCC*	miR-192-5p	Up		Side population cells (similar to CSC) vs. non-SP cells from HCC LM3 cells
[65]	HCC*	miR-192-5p	Down	CSC + HCCs vs. CSC- HCCs (including CD133 ± , EpCAM ± , CD44 ± , CD24 ± and CD90 ± . TCGA)	
[68]	Pancreatic cancer (PC)*	miR-192	Up	Serum of PC 74 patients vs. 29 type 2 diabetic patients or 17 HCs	
[69]	PC*	miR-192-5p	Down/up	PDAC tissues vs. ANT (down) Serum of PDAC UICC stage IV vs. HCs (up) Serum exosomes of PDAC UICC stages II to IV vs. HCs (up)	Gemcitabine-resistant cell clones of AsPC-1 vs. AsPC-1 (down) PDAC cells AsPC-1 and PANC-1 vs. human pancreatic duct epithelial cell line H6c7 (up)
[70]	PC*	miR-192	Up	10 PDACs tissues vs. 10 ANT Serum samples from 70 PDAC patients vs. 40 HCs	
[79]	Prostate cancer (PCa)*	miR-192	Up	PCa tissues vs. non-tumor tissues; metastatic samples vs. primary tumor tissues (TCGA、GEO)	
[80]	PCa*	miR-192	Down		prostate cancer lines PC-3 and DU145 vs. prostate epithelial RWPE-1 cells
[81]	PCa*	miR-192-5p	Up		combined treatment of BK002 (AJN and MFR) vs single AJN and MFR treatment in both PC3 and DU145 cells (castration-resistant prostate cancer cell line)

^*^ Expression of miR-192 has controversy

*ANT* adjacent non-cancerous tissue, *HCs* health controls, *NATs* non-tumor adjacent tissues, *AJN* Achyranthes japonica Nakai, *MFR* Melandrium firmum Rohrbach, *UICC* Union for International Cancer Control

### Upregulation of miR-192 in cancers

Several investigations have found elevated levels of miR-192 in various cancers, including cholangiocarcinoma, cervical cancer, hypoxic head and neck squamous cell carcinoma (HNSCC), and nasopharyngeal carcinoma. Specifically, studies have observed increased levels of miR-192-5p in cholangiocarcinoma tissues [12, 13], cell lines [12], urine [14], and serum of cholangiocarcinoma patients [15]. Similarly, elevated miR-192-5p levels have been found in cervical cancer tissues [16], hypoxic small extracellular vesicles (sEVs) from HNSCC [17], and nasopharyngeal carcinoma tissues and cells [18].

These findings emphasize the overexpression of miR-192 across various cancer types, indicating its potential role in cancer progression.

### Downregulation of miR-192 in cancers

The downregulation of miR-192 has been consistently reported across multiple cancer types, including breast cancer, glioma, medulloblastoma, colorectal cancer, bladder cancer, pediatric acute myeloid leukemia (AML), chronic lymphocytic leukemia (CLL), papillary thyroid carcinoma (PTC), osteosarcoma, and epithelial ovarian cancer (EOC) (Table 1). Numerous studies have documented decreased miR-192 expression in breast cancer tissues and cells [19, 20], with even lower levels observed in doxorubicin (DOX)-resistant breast cancer cell lines [21]. Significant reductions in miR-192 levels have also been observed in tissues and cells of glioma [22, 23], medulloblastoma [24], colorectal cancer [25–31], bladder Cancer [32], PTC [33], osteosarcoma [34–38], nephroblastoma subtypes [39], and endometrial cancer tissues [40], as well as in exosomes derived from tumor-associated macrophages (TAMs) [41].

Furthermore, miR-192 downregulation has been reported in various biofluids, including the serum of colon cancer [42, 43] and AML [44, 45] patients, the urine of bladder cancer patients [46] and the peripheral blood mononuclear cells (PBMCs) from CLL cases [47]. Interestingly, one study reported no difference in miR-192-5p expression in both colon cancer tissues and serum samples [43], suggesting further investigation to elucidate its role in this context.

### Variable expression of miR-192 in cancers

Certainly, the expression profile of miR-192 exhibits paradoxical patterns across different cancers, particularly in esophageal cancer, EOC, gastric cancer, hepatocellular carcinoma, pancreatic cancer, lung cancer, and prostate cancer.

miR-192 expression increases in the plasma of esophageal cancer patients [48] and in the tissues and cells of esophageal squamous cell carcinoma (ESCC) [49]. Additionally, miR-192 expression decreases post-therapy [50]. Notably, one study found that miR-192-5p expression rises following neoadjuvant chemotherapy and cisplatin treatment [48], suggesting its role in enhancing sensitivity to cisplatin, though it may also contribute to esophageal cancer progression.

In EOC, miR-192 has been shown to be highly expressed in ovarian mucinous carcinoma tissues [51] but reduced in plasma exosomes of individuals with EOC [52]. This indicates that cancer development involves diverse types and phases, each characterized by distinct molecular features that contribute to the variation in miR-192 expression. Even within the same cancer, distinct cell types can exhibit multiple miR-192 expression patterns. For instance, miR-192 expression is higher in the serum of gastric cancer patients with distant metastasis compared to those without metastasis [67]. Additionally, studies reported both upregulation [53–58] and downregulation [43, 59, 60] of miR-192 in gastric cancer tissues, cell lines, and serum from gastric cancer patients, which indicates significant variability in its expression.

A similar pattern of discrepancies is observed in hepatocellular carcinoma and pancreatic cancer. In hepatocellular carcinoma, miR-192 is generally downregulated in tissues [61–64] and cell lines [62, 65, 66], but upregulated in patients’ serum exosomes and specific cell populations resembling cancer stem cells (CSC) [67]. In pancreatic cancer, elevated expression of miR-192 has been observed in the serum of patients [68], as well as in serum exosomes, tissues, and pancreatic ductal adenocarcinoma (PDAC) cells [69, 70]. However, it is downregulated in PDAC tissues and gemcitabine-resistant cells [69]. These variations can be attributed to the diverse sources of circulating miRNAs, which include tumor cells and PBMCs. miRNAs are encapsulated within extracellular vesicles or bound to protein complexes, providing protection from degradation and explaining the discrepancies in miR-192 levels between tissues and circulation. Furthermore, the differing half-lives of miRNAs in blood versus tissues, influenced by factors such as RNase activity, contribute to these variations.

In lung cancer, miR-192 displays diminished expression in tissues [71], serum of patients with bone metastasis [72], and bronchial lavage fluid [73]. However, cellular studies present conflicting results, with some showing decreased expression [72, 73], while others report increased expression [74–76], particularly higher in cisplatin-resistant lung carcinoma cells [77, 78]. In prostate cancer, data from the Cancer Genome Atlas (TCGA) and Gene Expression Omnibus (GEO) show elevated miR-192 expression in tissues [79], whereas cell studies on cultured cells report downregulation [80, 81]. Notably, cell lines often deviate from the characteristics of the original tumor tissues due to accumulated mutations over time, which can lead to discrepancies in miRNA expression. Additionally, the tumor microenvironment and various cell types present in tumor tissues further influence miR-192 levels, which may not be fully represented by in vitro studies.

Therefore, miR-192 shows variable expression across different cancers, influenced by factors such as tumor stage, metastasis, drug resistance, and the tumor microenvironment. These expression differences highlight its potential as a biomarker for cancer diagnosis, prognosis, and treatment monitoring. However, further research is needed to fully elucidate its role and mechanisms in different cancer types.

## The role of miR-192 in cancer progression

**Fig. 1:**
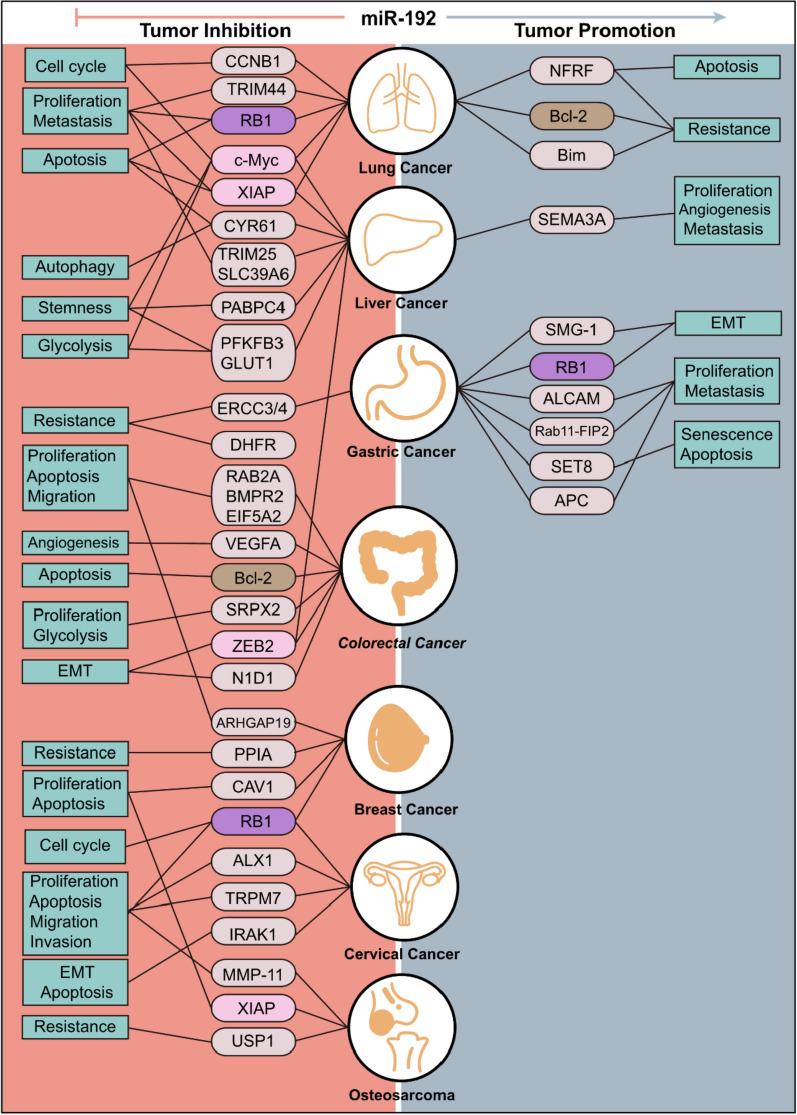
Pathological Processes Involving miR-192 in Tumorigenesis. This figure illustrates the dual roles of miR-192 across cancer types, where it functions either as a tumor suppressor or an oncogene by targeting specific genes

The dysfunction of miR-192 is implicated in several key processes of tumorigenesis, including cancer cell proliferation, apoptosis, migration, invasion, metastasis, EMT, and angiogenesis. miR-192 exerts distinct roles across various tumors by regulating multiple target genes. Its primary role in most cancers is to prevent tumor development, However, in gastric cancer, it predominantly exhibits oncogenic properties (Fig. 1). The tumor-suppressive effect of miR-192 on specific target genes, such as *MYC*, *XIAP*, and *ZEB2*, is consistent across various cancers (Fig. 1). However, miR-192 can exhibit dual effects when targeting the same gene in different cancers, such as *BCL-2* and *RB1* (Fig. 1). Therefore, the distinct functions of miR-192 can be further explored by targeting specific genes in distinct types of cancer (Fig. 1).

### Gastric cancer

Most studies have found that miR-192 functions as an oncogene in gastric cancer. Specifically, miR-192 has been observed to enhance cell proliferation and migration by downregulating *ALCAM* [56], *APC* [55], and *Rab11-FIP2* [82]. In addition, it promotes EMT and Treg cell differentiation by suppressing *RB1* [53]. Its role in decreasing SET domain containing lysine methyltransferase 8 (SET8) expression prevents cellular senescence and apoptosis, thus promoting gastric cancer initiation and progression [54].

However, one specific study identifies miR-192-5p as a tumor suppressor, reversing cisplatin resistance in gastric cancer cells by targeting *ERCC3* and *ERCC4* [60].

### Lung cancer

Lung cancer remains consistently ranked as the most diagnosed cancer and was the primary cause of cancer-related deaths by 2022 [1, 2]. miR-192 plays a complex role in lung cancer, acting both as a tumor suppressor and an oncogene. It induces cell cycle arrest by downregulating *CCNB1* and *MYC* [83, 84]. It also inhibits proliferation and EMT while promoting apoptosis through *RB1* downregulation in non-small-cell lung cancer (NSCLC) [71, 85], and has been found to target *TRIM44* to curb cancer cell proliferation, migration, and invasion [54]. Moreover, miR-192-5p exerts pro-apoptotic effects by interacting with *XIAP* in NSCLC cells [56].

Additionally, miR-192 may contribute to lung cancer progression by promoting cisplatin resistance and inhibiting apoptosis through the downregulation of *NKRF* [77] or *BIM* [78]. Additionally, miR-192 regulates chemoresistance to combined gemcitabine and cisplatin chemotherapy by targeting *BCL-2* in human adenocarcinoma lung cancer A549 cells [74].

### Liver cancer

Primarily, miR-192 primarily functions as a tumor suppressor in liver cancer. Studies have shown that miR-192-5p triggers apoptosis and hampers cell growth, migration, and invasion in hepatocellular carcinoma by downregulating *XIAP* [86]. Additionally, miR-192 targets *TRIM25* to inhibit proliferation and migration in hepatitis B virus-related hepatocellular carcinoma [61], and targets *SLC39A6* to reduce tumor metastasis in hepatocellular carcinoma cells [62]. Upregulation of miR-192-5p has also been associated with the activation of autophagy and apoptosis via *CYR61* in hepatocellular carcinoma [66].

Interestingly, the absence of miR-192-5p has been observed to promote stemness features in CSC-positive hepatocellular carcinoma cells by upregulating *MYC*, *PFKFB3*, *GLUT1* [87], and *PABPC4* [65]. Another study demonstrated that *ZEB2*, both a target of miR-192 and an EMT activator, increases in the absence of miR-192, thereby promoting EMT and invasion [88].

However, a few studies indicate the oncogenic role of miR-192. For instance, miR-192-5p has been implicated in targeting *SEMA3A*, potentially enhancing cell proliferation, angiogenesis, and metastasis [67].

### Colorectal cancer

Colorectal cancer research has identified miR-192-5p as a tumor suppressor, playing a crucial role in inhibiting cancer progression. In particular, miR-192-5p modulates apoptosis by targeting *EIF5A2* [30], *RAB2A* [26], and *BMPR2* [25], while also inhibiting cell proliferation, migration, and invasion. Additionally, its interaction with *SRPX2* has been shown to suppress colorectal cancer development by inhibiting proliferation and enhancing glycolysis [28]. It also promotes apoptosis by downregulating *BCL-2* [29], and inhibits EMT by targeting *ZEB2* [29] and *NID1* [89]. In addition, it hinders angiogenesis by regulating *VEGFA* [29] in colorectal cancer. Another study demonstrated that miR-192 sensitizes HCT-116 cells to methotrexate (MTX) by regulating *DHFR*, suggesting a novel therapeutic approach to the treatment of patients with colon cancer [90].

### Breast cancer

Breast cancer is the most commonly diagnosed cancer among women and is the leading cause of cancer-related deaths globally [2]. In breast cancer, miR-192 exhibits tumor-suppressive effects. Specifically, miR-192 inhibits proliferation and promotes apoptosis by targeting *CAV1* [19]. Additionally, miR-192-5p sensitizes breast cancer cells to DOX and regulates cell growth by targeting *PPIA*, highlighting its potential as a therapeutic target [21]. A mechanistic study further demonstrated that miR-192 induces cell cycle arrest by downregulating *RB1* [20]. In triple-negative breast cancer (TNBC), miR-192 also acts as a tumor suppressor by increasing apoptosis and inhibiting both proliferation and migration, with *ARHGAP19* identified as a novel target gene of miR-192 [91].

### Cervical cancer

Cervical cancer ranked fourth for both incidence and mortality among women by 2022 [2]. In cervical cancer, miR-192-5p exerts a tumor-suppressive function by inhibiting proliferation, migration, and invasion through direct targeting of *TRPM7* [92]. Silencing miR-192-5p has the opposite effect, promoting these oncogenic processes by regulating *ALX1* [40] and *RB1* [93]. Furthermore, miR-192-5p derived from TAMs exosomes has been observed to suppress EMT and promote apoptosis by targeting *IRAK1* in endometrial cancer [41].

### Osteosarcoma

miR-192 plays a tumor-suppressive role in osteosarcoma by regulating cell proliferation, apoptosis, migration, and invasion. It achieves these effects by selectively targeting specific genes, including *TCF7* [37], *MMP11* [35], and *USP1* [36]. Additionally, another study demonstrated that miR-192 inhibits osteosarcoma cell proliferation and induces apoptosis by interacting with *XIAP* [34]. Additionally, miR-192 has been shown to enhance the sensitivity of MG-63 cells to MTX [94] and to improve the sensitivity to cisplatin by targeting *USP1* [36].

### Others

In nasopharyngeal carcinoma, miR-192 promotes tumor progression through *RB1* inhibition [18], while in HNSCC, it enhances malignancy via *CAV1* downregulation [17]. Similarly, miR-192 contributes to neuroblastoma development by targeting *Dicer1* [95].

Conversely, miR-192 serves a tumor-suppressive function in various cancers. In PTC, it reduces malignancy by regulating *SH3RF3*, affecting migration, invasion, and EMT [33]. In bladder cancer, miR-192 inhibits tumor growth [32, 96], by downregulating the transcription factor Yin Yang 1 linked to cancer cell proliferation [32], and is also associated with gemcitabine resistance in bladder cancer [97]. In multiple myeloma, miR-192 acts as a tumor suppressor by targeting IL-17Rs [98], and bioinformatic analyses suggest it connects with the bone marrow microenvironment by interacting with *CDKN2A*, influencing tumor progression [99]. In AML, miR-192 suppresses cancer progression by targeting *CCNT2* [45], *ULK1* [100], and *ZBTB20* [101], with additional anti-tumor effects linked to modulation of the WNT signaling pathway [102].

In renal cancers, miR-192 acts as a tumor suppressor by targeting *MDM2*, *ZEB2*, and *TYMS* in renal cell carcinoma, thereby reducing migration and invasion [103]. In nephroblastoma, it inhibits tumor growth by downregulating *ACVR2B* [39], and it further limits angiogenesis in renal tumors through targeting *ECR1* [104]. Additionally, miR-192 suppresses tumor growth in brain cancers, targeting *RAB2A* in glioblastoma multiforme [23], *ZEB2* in glioma [22], and *DHFR* in medulloblastoma [24]. In retinoblastoma, miR-192 curtails tumor cell migration and invasion by regulating *ITGA1* [105].

In ovarian cancer, miR-192 exhibits anti-angiogenic effects by targeting *HOXB9* [104]. Bioinformatic studies also identify miR-192-5p as a key miRNA in high-grade primary ovarian tumors [106].

Notably, miR-192 displays a dual role in pancreatic, esophageal, and prostate cancers. In PDAC, miR-192 overexpression promotes tumor growth by repressing *SIP1* [70], though other studies suggest it may function as a tumor suppressor by inhibiting EMT via *ZEB2* downregulation [69]. In esophageal cancer, miR-192 enhances cisplatin sensitivity by targeting *ERCC3/4* [48] and increases tumor cell susceptibility to cytotoxic T lymphocytes by targeting *BCL-2* [107]. However, miR-192 promotes proliferation and inhibits apoptosis by suppressing *BIM* [49], indicating a complex role in esophageal cancer progression and treatment response. In prostate cancer, miR-192 reduces cell proliferation by targeting *NOB1* [80], though contrasting findings indicate it may also promote cell cycle progression and proliferation [79].

## Mechanism of action of miR-192 as a tumor suppressor

**Fig. 2:**
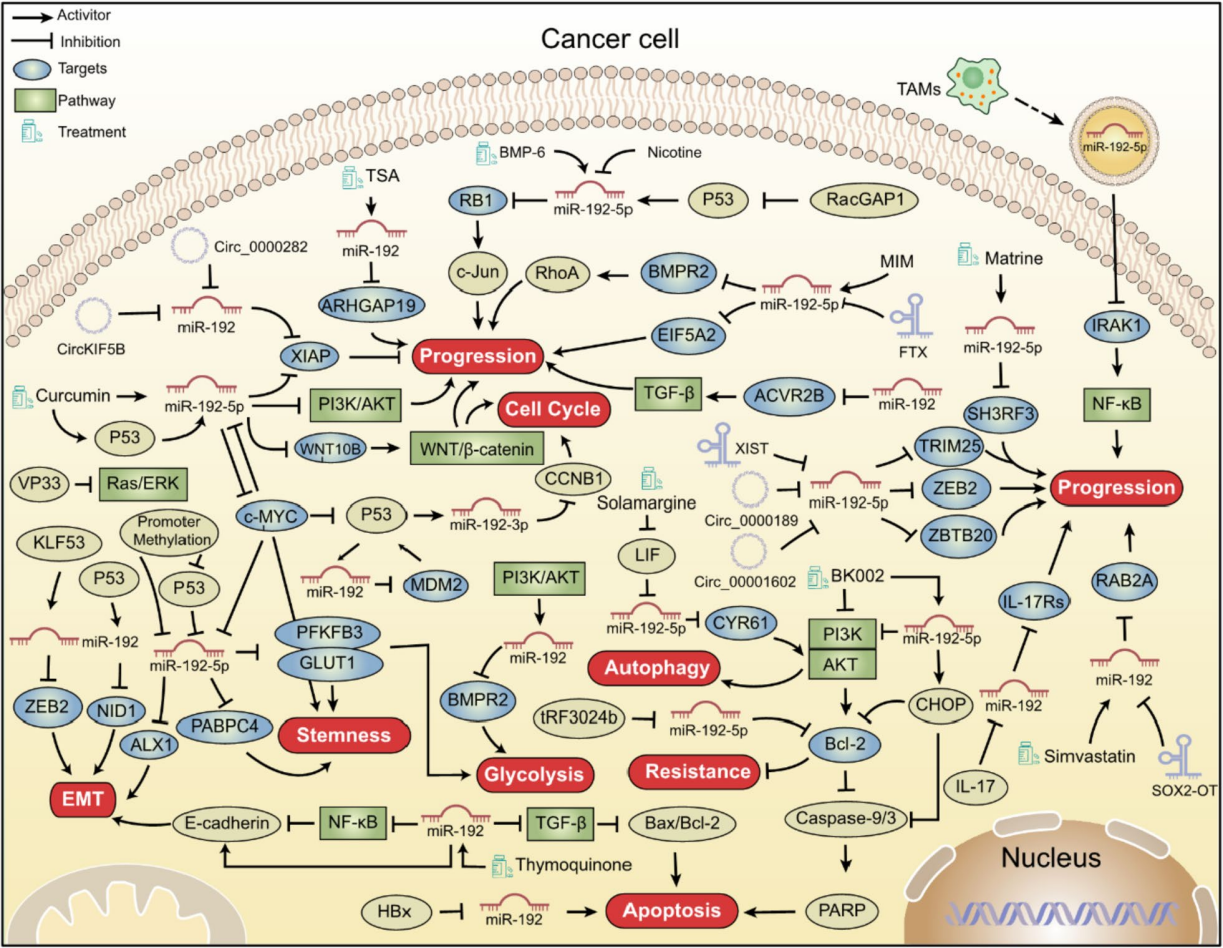
The Anti-tumor Mechanisms of miR-192 in Cancer. This figure provides a comprehensive summary of the regulatory pathways through which miR-192 exerts tumor-suppressive effects in various cancers, including its upstream regulators, downstream targets, and associated signaling pathways, as well as potential therapeutic implications

Primarily, miR-192 acts as a tumor suppressor in cancer by regulating specific genes and pathways (Fig. 2). miR-192 is regulated by upstream factors, including non-coding RNAs, pharmaceutical treatments, and other factors.

### Regulation of miR-192 by upstream factors

Circular RNAs (circRNAs) and long non-coding RNAs (lncRNAs) regulate cancer progression by acting as competitive endogenous RNAs (ceRNAs). They sequester miR-192, thereby protecting mRNAs from miRNA-induced degradation [108, 109]. For instance, circKIF5B [86] and circ_0000282 [34] promote osteosarcoma progression by specifically modulating the miR-192/*XIAP* axis. Similarly, circ_0000189 and circ_0001602 facilitate glioma and AML progression via the miR-192-5p/*ZEB2* [22] and miR-192-5p/*ZBTB20* axes, respectively [101].The IncRNAs such as SOX2-OT [23], XIST [61], and FTX [30] enhance the production of *RAB2A*, *TRIM25*, and *EIF5A2* by sponging miR-192, thereby contributing to glioblastoma multiforme, hepatocellular carcinoma, and colorectal cancer progression, respectively.

Several pharmacological agents target miR-192 to regulate cancer progression, offering promising therapeutic strategies. For example, thymoquinone preserves miR-192 levels, inhibiting EMT by increasing E-cadherin expression and inducing apoptosis through the BAX/BCL-2 ratio [64]. BK002, a compound derived from *Achyranthes japonica Nakai* (AJN) and *Melandrium firmum Rohrbach* (MFR), both of which have been traditionally used as herbal medicines in China and Korea, induces the expression of miR-192-5p and the C/EBP homologous protein (CHOP). This in turn triggers apoptosis by downregulating BCL-2 and upregulating caspase activity [81]. Simvastatin activates miR-192 to inhibit colon cancer progression by targeting *RAB2A* [26]. Additionally, curcumin-induced upregulation of miR-192 in NSCLC exhibits anti-proliferative effects by targeting *MYC* and induces apoptosis by targeting *XIAP* [75, 84, 110]. Additionally, solamargine that is a natural compound found in *Solanum nigrum L*., exhibits multifaceted antitumor mechanisms. It elevates miR-192-5p expression through leukemia inhibitory factor (LIF) regulation, effectively inducing autophagy and apoptosis in hepatocellular carcinoma by targeting *CYR61* [66]. Matrine, a quinolizidine alkaloid extracted from the traditional Chinese medicinal plant *Sophora flavescens*, exhibits diverse biological activities, including anticancer, anti-inflammatory, and antiviral effects. In thyroid cancer cells, matrine inhibits migration and invasion by regulating the miR-192-5p/*SH3RF3* signaling axis [33].

Other factors also regulate miR-192 expression across various cancers. Nicotine downregulates miR-192 in NSCLC, enhancing proliferation and EMT through *RB1* upregulation [85]. Rac GTPase-activating protein 1 (RacGAP1) inhibits miR-192-5p expression by suppressing p53, thereby promoting the carcinogenesis of cervical cancer through *RB1* overexpression [93]. The intestinal microflora (MIM) in colon cancer increases miR-192-5p levels, inhibiting progression through *BMPR2* suppression [25]. Furthermore, a regulatory feedback loop involving IL-17, miR-192, and IL-17Rs regulates proliferation and EMT in multiple myeloma [98]. tRF-3024b, a specific tRNA-derived fragment, hijacks miR-192-5p in ESCC, enhancing BCL-2 levels and contributing to cytotoxic T lymphocyte resistance [107]. Additionally, a study proposed a model in which p53 typically binds to miR-192, but when p53 binding is lost (due to *P53* loss or mutation), Kruppel-like factor 5 (KLF5) binds to miR-192 instead [88]. This interaction leads to the transactivation of miR-192, effectively substituting for p53 function and inhibiting EMT in liver cancer cells. However, the loss of KLF5 results in the inactivation of miR-192 transcription. The expression of *ZEB2*, a downstream target of miR-192 and a driver of EMT, regulates EMT and invasion in cancers.

### Pathways

#### PI3K/AKT signaling pathway

The PI3K/AKT signaling pathway is a crucial cellular signaling cascade involved in processes such as cell survival, proliferation, growth, and metabolism. Dysregulation of this pathway is associated with the development of several diseases, particularly cancer. Curcumin has shown the ability to inhibit cell proliferation and induce apoptosis in NSCLC cells. These effects are attributed to the upregulation of miR-192-5p, which suppresses the PI3K/AKT signaling pathway [75]. Similarly, BK002 has been shown to hinder the PI3K/AKT signaling pathway by upregulating miR-192, thereby promoting apoptosis in castration-resistant prostate cancer [81]. Furthermore, solamargine has been found to enhance cell apoptosis and autophagy by reducing the phosphorylation of AKT (pAKT) which is a key component of the PI3K/Akt pathway [66].

#### NF-κB and MAPK signaling pathways

The Nuclear Factor-kappa B (NF-κB) signaling pathway is a pivotal cellular pathway that regulates the transcription of genes involved in immune responses, inflammation, cell survival, and proliferation. In normal cells, NF-κB remains inactive in the cytoplasm by binding to IκB [111]. Upon IκB degradation, NF-κB translocates into the nucleus to activate target genes and execute its biological functions [111]. This pathway is implicated in various pathological processes in cancer. Thymoquinone has been shown to protect liver tissues by preserving miR-192 and interrupting the NF-κB signaling [64]. Additionally, the overexpression of miR-192-5p in TAMs-derived exosomes exhibits a suppressive effect on EMT by inhibiting the NF-κB signaling pathway in esophageal cancer [41].

The Mitogen-Activated Protein Kinase (MAPK) pathway is a fundamental signaling cascade that mediates the transmission of extracellular signals to the nucleus. This pathway regulates a spectrum of cellular processes, including cell growth, proliferation, differentiation, survival, and response to stress. This pathway plays a pivotal role in coordinating cellular responses to various stimuli, including growth factors, cytokines, and environmental stressors. Furthermore, Vacuolar Protein Sorting 33B (VPS33B) has been shown to increase the expression of miR-192-3p through the inactivation of Ras/ERK signaling. This leads to the inhibition of *CCNB1* and subsequent blockade of cell proliferation [83].

#### TGF-β/SMAD and WNT/β-catenin signaling pathways

The Transforming Growth Factor-beta (TGF-β) signaling pathway is a pivotal and multifunctional cascade that intricately regulates diverse cellular processes, including cell proliferation, differentiation, apoptosis, and immune responses. It plays a crucial role in embryonic development, tissue homeostasis, and maintaining immune system balance. Dysregulation of the TGF-β pathway has been linked to various diseases, including cancer and fibrosis. Thymoquinone protects liver tissues by maintaining the levels of miR-192 and E-cadherin while also inhibiting TGF-β signaling [64]. Additionally, *ACVR2B*, which encodes a protein that functions as a member of the TGF-β signaling pathway, has been identified as a target gene of miR-192 [39]. The downregulating miR-192 may contribute to nephroblastoma development by targeting *ACVR2B* [39]. These findings emphasize the intricate role of the TGF-β signaling pathway in physiological processes and its involvement in pathological conditions.

The Wnt/β-catenin signaling pathway, also known as the classical Wnt pathway, is a critical and evolutionarily conserved cellular signaling cascade with essential roles in embryonic development, tissue homeostasis, and disease. Dysregulation of this pathway is implicated in multiple cancers. The inhibitory effects of curcumin on NSCLC cells may be associated with the upregulation of miR‑192‑5p. This occurs through the targeting of *MYC* and the deactivating of Wnt/β-catenin signaling pathway [84]. Delivery of miR-192 via hydrogel-based methods inhibits hepatocellular carcinoma progression by specifically targeting *WNT10B*, encoding Wnt Family Member 10B protein (WNT10B) which plays a crucial role in the GSK3β/Wnt/β-catenin pathway [112]. This finding highlights the complex role of the Wnt/β-catenin pathway in cellular processes and its potential modulation for therapeutic purposes, particularly involving miRNAs such as miR‑192‑5p.

These intricate regulatory mechanisms underscore the multifaceted role of miR-192 in cancer pathogenesis and underscore its potential as a therapeutic target in diverse malignancies.

## Mechanism of action of miR-192 as an oncogene

**Fig. 3:**
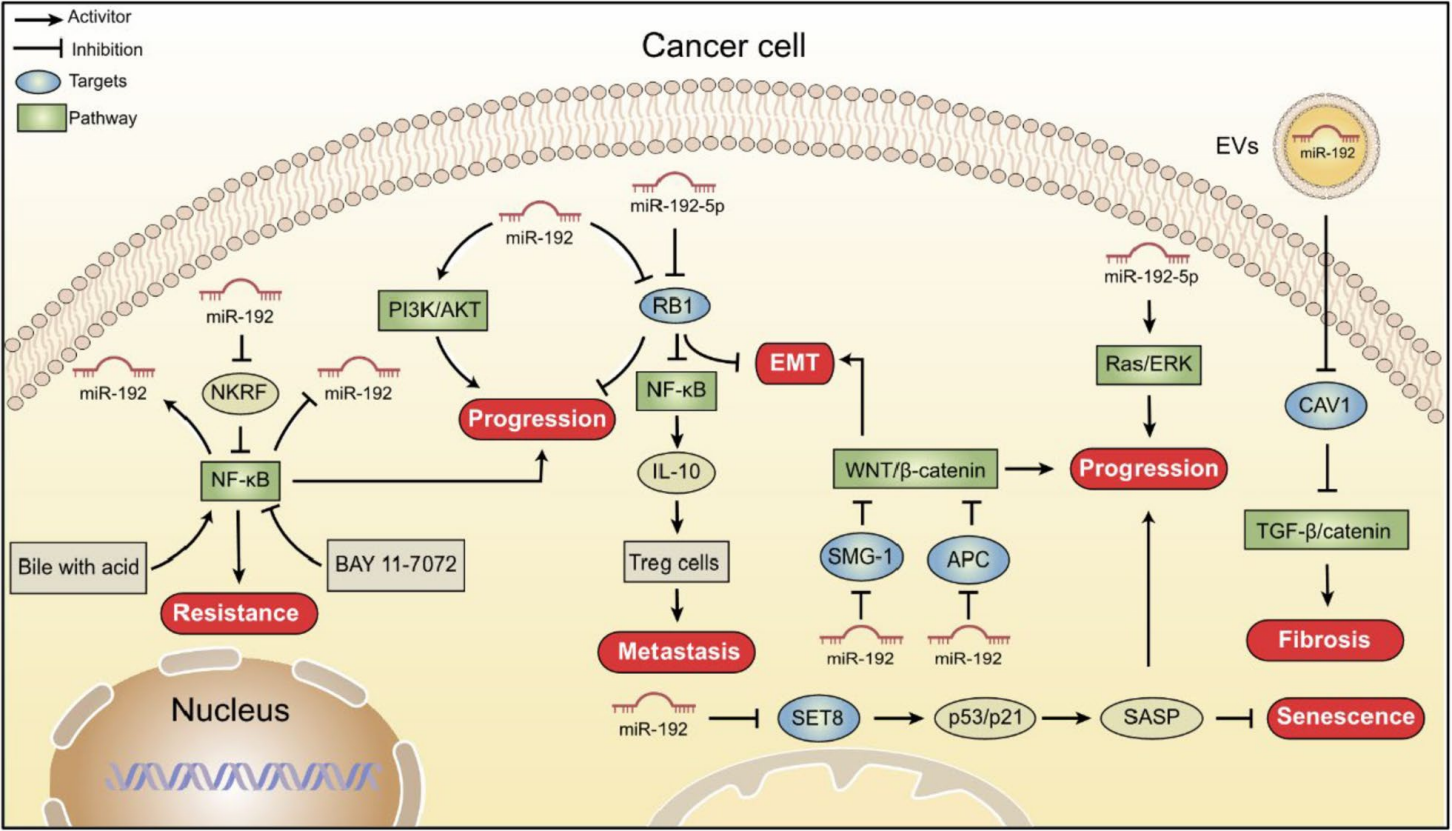
The Oncogenic Mechanisms of miR-192 in cancer. This figure presents an overview of the regulatory pathways and molecular interactions through which miR-192 functions as an oncogene in various cancers, highlighting its contribution to tumor progression and malignancy

While miR-192 acts as a tumor suppressor in many cancers, its role as an oncogene has also been observed across different malignancies. The regulation of miR-192 expression and activity in cancer is highly complex, involving multiple factors and signaling pathways (Fig. 3).

### Regulation of miR-192 by upstream factors

The combination of bile and acid induces the deregulation of miR-192, a process facilitated by NF-κB activation, is associated with molecular and early histopathological alterations linked to neoplastic transformation [113]. Similarly, the NF-κB inhibitor BAY 11–7072 effectively prevents the acidic bile-induced upregulation of miR-192 in normal human hypopharyngeal cells [114].

### Pathways

#### NF-κB and WNT/β-catenin signaling pathways

miR-192 can activate NF-κB either in various cancers by either directly or indirectly influencing specific target genes. In gastric cancer, miR-192 activates NF-κB by repressing its target *RB1*, hence promoting tumorigenesis through the induction of EMT [53]. Similarly, miR-192 downregulates the target gene *NKRF* to directly activate NF-κB, which contributes to tumorigenesis by inhibiting cell apoptosis, enhancing cell cycle progression, and promoting chemo-resistance in lung cancer [77]. Additionally, acidic bile induces upregulation of miR-192 by indirectly activating NF-κB, thereby promoting tumorigenesis in laryngeal cancer [113]. Conversely, the NF-κB pathway inhibitor BAY 11–7072 reduces the expression of miR-192 induced by acidic bile [114].

The WNT signaling pathway is activated by tumor suppressor *APC* [55] and *SMG-1* [115], which have been identified as target genes of miR-192. This activation contributes to the occurrence and development of gastric cancer.

#### MAPK and PI3K-Akt signaling pathways

miR-192-5p has been shown to promote proliferation and suppress apoptosis in cholangiocarcinoma cells by activating the MEK/ERK pathway [12]. Additionally, miR-192 can activate the PI3K/Akt pathway, contributing to the progression of nasopharyngeal carcinoma [18].

## Clinical application of miR-192 in human cancers

In cancer research, miR-192 has emerged as a central focus due to its potential as a diagnostic or prognostic biomarker across various cancer types. Its detectability in various biological fluids and tissues, and resistance to degradation by endogenous ribonuclease, make miR-192 an economical, sensitive, and non-invasive biomarker method. This overview explores the diverse value of miR-192 in cancer diagnosis and prognosis (Table 2).

**Table 2:** Recent clinical applications of miR-192

Refs	Disease	Origin	Samples	Tendency	Clinical Application
[13]	Cholangiocarcinoma (CCA)	Serum	94 patients with cholangiocarcinoma undergoing tumour resection and 40 healthy controls	Up	Diagnosis and prognosis
[14]	CCA	Urinary	Urinary miR-192 level in healthy subjects (n = 21), O. viverrini-infected subjects (n = 24), subjects with periductal fibrosis (n = 28), CCA patients (n = 22) and patients with other inflammatory diseases (n = 10)	Up	Diagnosis
[15]	CCA	Serum	Serum from 11 CCA patients and nine healthy subjects	Up	Prognosis
[44]	Pediatric AML	Serum	The serum from 97 AML cases and 50 healthy controls	Down	Diagnosis and prognosis
[47]	CLL	PBMCs	PBMCs of CLL patients (n = 20) in comparison with healthy subjects (n = 20)	Down	Diagnosis and prognosis
[119]	Hepatocellular carcinoma (HCC)	Plasma exosome	Exosomes derived from the plasma of 24 (14 early and 10 advanced) HCC patients, 37 liver cirrhosis patients and 20 HCs Exosomal levels from the plasma of 84 HCC patients, 50 liver cirrhosis patients, and 20 HCs	Up	Diagnosis and prognosis
[42]	Colorectal cancer (CRC)	Serum	The serum of 164 colon cancer patients and 60 healthy controls	Down	Diagnosis and prognosis
[27]	CRC	Tissue	16 Stage IIIB patients (8 patients with liver metastasis between 8 and 18 months after radical operation to remove their colon cancers.) and control (8 patients: 4 right colon cancer patients and 4 left colon cancer patients) groups	Down	Prognosis
[31]	CRC	tissue	107 pairs of CRC tissues and non-tumor adjacent tissues	Down	Diagnosis
[52]	EOC	Plasma exosome	Plasma exosomes were isolated from peripheral blood of EOC patients and healthy donors	Down	Diagnosis and prognosis
[51]	Ovarian carcinoma	Tissue	89 samples (53 high-grade serous carcinomas, 17 endometrioid carcinomas, 7 low-grade serous carcinomas, 5 clear cell carcinomas, 4 borderline tumors, and 3 mucinous carcinomas)	Up (mucinous) Down (others)	Diagnosis
[57]	Gastric cancer (GC)	Plasma exosome	The exosomal microRNAs (ex-miRNAs) were isolated from plasma samples from 60 gastric cancer (GC) patients and 63 healthy individuals	Up	Diagnosis and prognosis
[43]	GC	Tissue	25 samples of gastric cancer and ANTs	Down	Diagnosis
[58]	GC	Plasma	Plasma levels in GC/DM samples (n = 48) compared with GC/NDM (n = 48) and HCs (n = 30)	Up	Diagnosis
[59]	GC	Tissue	118 pairs of gastric cancer tissues and non-tumor counterparts (as controls)	Down	Diagnosis
[69]	PDAC	Tissue, serum, plasma exosome	26 PDAC tissues vs 19 HCs and 26 ANTs serum of PDAC UICC stage IV vs. 19 HCs Serum exosomes of PDAC UICC stages II to IV vs. HCs	Down (tissues) Up (serum, exosome)	Diagnosis and prognosis
[68]	Pancreatic cancer (PC)	Serum	In serum of PC 74 patients with PC, 29 type 2 diabetic patients and 17 controls	Up	Diagnosis
[70]	PDAC	tissue, serum	Pooled tissue extracts of 10 PDACs and adjacent normal controls (n = 10) Serum samples from PDAC patients (n = 70) compared to duodenal adenocarcinoma patients (n = 17) and healthy controls (n = 40)	Up	Diagnosis
[16]	Cervical cancer	Tissue	40 clinical cervical tissue samples containing 20 normal (Norm) and 20 adenocarcinoma (Tumor) tissue samples	Up	Diagnosis
[79]	prostate cancer (PCa)	Tissue	PCa tissues compared to nontumor tissues; metastatic samples compared to primary tumor tissues (TCGA、GEO)	Up	Diagnosis
[17]	HNSCC	Plasma exosome	In hypoxic sEVs compared with normoxic sEVs	Up	Diagnosis
[46]	Bladder cancer	Urine	Morning urine from bladder cancer (n = 118) and benign urinary system diseases (control, n = 120)	Down	Diagnosis
[73]	NSCLC	Bronchial lavage	Bronchial lavage samples from NSCLC participants and HCs (n = 100)	Down	Prognosis
[38]	Osteosarcoma	Tissue	80 pairs of osteosarcoma and corresponding noncancerous bone tissues	Down	Prognosis
[122]	Larynx/HSCC	Tissue	Tumors of patients with late/no recurrence vs. patients with early recurrence	Down	Prognosis
[50]	Esophageal cancer (EC)	Tissue	80 post-therapeutic samples of EC compared to corresponding pretherapeutic samples	Down	Prognosis

*HCs* health controls, *ANTs* adjacent non-cancerous tissues, *GC/DM* gastric cancer with distant metastasis, *GC/NDM* gastric cancer with no distant metastasis, *UICC* Union for International Cancer Control

### Diagnostic biomarker

Firstly, miR-192 exhibits significant diagnostic potential across multiple cancer types, with its expression in blood (including serum, serum exosomes and PBMCs), tissues, and urinary sediment emerging as a valuable diagnostic marker.

In pancreatic cancer (including PDAC) [68–70, 116–118] and gastric cancer [43, 57–59], miR-192 serves as a reliable biomarker in serum, serum exosomes and tissue samples. Similarly, its presence in serum and tissue from colorectal cancer patients enhances its diagnostic utility [31, 42]. Also, miR-192 levels in hepatocellular carcinoma [119], EOC [52], and HNSCC [17], particularly in serum and serum exosomes, underline its broader clinical applicability. Additionally, circulating miR-192 in serum has shown promise as a marker for the early detection of cholangiocarcinoma [13], multiple myeloma [120] and pediatric AML [44]. Furthermore, mR-192 expression in PBMCs also holds potential for the early diagnosis of CLL [47].

miR-192 expression in tissues has shown diagnostic value in cervical cancer, prostate cancer, and ovarian carcinoma. Notably, miR-192 levels were observed to be six times higher in mucinous tumors compared to other histotypes of ovarian carcinoma [51]. This distinct pattern underlines the potential of miR-192 as a diagnostic biomarker, particularly for mucinous tumors within the spectrum of ovarian carcinoma.

Furthermore, miR-192 levels in urine have emerged as diagnostic biomarkers for bladder cancer [46, 121] and liver fluke-associated cholangiocarcinoma [14]. A decrease of miR-192 expression in the urinary sediment from bladder cancer patients may indicate tumor progression and combining miR-192 expression in urinary sediment with B-ultrasound demonstrates high sensitivity in diagnosing bladder cancer [46].

### Prognostic biomarker

Also, miR-192 has demonstrated significant prognostic value across various cancer types, with its expression correlating with disease progression and clinical outcomes. In cholangiocarcinoma, postoperative declines in circulating miR-192 are associated with favorable outcomes, including reduced lymph node metastasis and improved overall survival [13, 15]. Similarly, in NSCLL, miR-192 expression levels in serum and bronchial wash samples are significantly associated with TNM stages, distant metastases, pathological grade, and overall survival, establishing its role as a valuable prognostic marker [73]. In colon cancer, miR-192 levels in serum and tissue samples from colon cancer patients have been linked to poor differentiation, lymphatic metastasis, vascular invasion, and advanced TNM stages, further underscoring the prognostic value of miR-192 [27, 31, 42, 43]. Reduced miR-192 expression in serum from pediatric AML patients has been associated with poor prognosis [44], while miR-192 levels in PBMCs hold potential as a prognostic biomarker for CLL [47]. Exosomal miR-192 also exhibits prognostic significance in hepatocellular carcinoma [119], EOC [52] and gastric cancer [57], where its overexpression correlates with poor overall survival. Likewise, miR-192 expression patterns in colorectal cancer, osteosarcoma, HNSCC, and esophageal cancer tissues correlate with clinical outcomes, offering further prognostic insights [38, 50, 122].

## Conclusions

A substantial body of research has explored the context-dependent roles of miR-192 in various cancers, underscoring its promising clinical potential. The distinct expression patterns of miR-192 across cancer types highlight its value as a diagnostic and prognostic biomarker. Notably, aberrant miR-192 levels detected in whole blood, plasma, and other patient specimens suggest its potential utility in non-invasive cancer diagnosis, disease monitoring, and prognostic assessment. Also, miR-192 plays a critical role in the molecular networks underlying tumorigenesis by modulating key functional pathways. These findings highlight miR-192 as potential therapeutic targets, offering new avenues for cancer treatment and intervention. Recent advancements in delivery technologies, including nanoparticles, liposomes, and viral vectors, have improved the feasibility of miRNA-based therapies by enabling precise delivery to tumor sites or systemic administration. Non-invasive delivery strategies are also under development to enhance miRNA transport across cellular barriers, improving targeting specificity and therapeutic efficiency.

This review has comprehensively discussed the aberrant expression, diverse functions, and clinical applications of miR-192 in cancer, with an emphasis on its regulatory mechanisms and networks. However, many of the mechanisms and pathways highlighted are based on preliminary findings, necessitating further validation through larger-scale studies to confirm their clinical relevance and therapeutic applicability.

## Data Availability

No datasets were generated or analysed during the current study.

## Abbreviations

miR-192: microRNA-192
miRNAs: microRNAs
EMT: Epithelial-mesenchymal transition
HNSCC: Hypoxic head and neck squamous cell carcinoma
sEVs: Small extracellular vesicles
AML: Acute myeloid leukemia
CLL: Chronic lymphocytic leukemia
PTC: Papillary thyroid carcinoma
EOC: Epithelial ovarian cancer
DOX: Doxorubicin
TNBC: Triple-negative breast cancer
PBMCs: Peripheral blood mononuclear cells
TAMs: Tumor-associated macrophages
ESCC: Esophageal squamous cell carcinoma
CSC: Cancer stem cell
PDAC: Pancreatic ductal adenocarcinoma
NSCLC: Non-small-cell lung cancer
MTX: Methotrexate
circRNAs: Circular RNAs
lncRNAs: Long non-coding RNAs
NF-κB: Nuclear Factor-kappa B
MAPK: Mitogen-Activated Protein Kinase
TGF-β: Transforming Growth Factor-beta

## References

[1] Sung H, Ferlay J, Siegel R, Laversanne M, Soerjomataram I, Jemal A, Bray F. Global Cancer Statistics 2020: GLOBOCAN estimates of incidence and mortality worldwide for 36 cancers in 185 Countries. CA A Cancer J Clin. 2021;71(3):209–49. 10.3322/caac.21660 33538338

[2] Bray F, Laversanne M, Sung H, Ferlay J, Siegel RL, Soerjomataram I, Jemal A. Global cancer statistics 2022: GLOBOCAN estimates of incidence and mortality worldwide for 36 cancers in 185 countries. CA A Cancer J Clin. 2024;74(3):229–63. 10.3322/caac.21834 38572751

[3] Condrat CE, Thompson DC, Barbu MG, Bugnar OL, Boboc A, Cretoiu D, Suciu N, Cretoiu SM, Voinea SC. miRNAs as biomarkers in disease: latest findings regarding their role in diagnosis and prognosis. Cells. 2020;9(2):276. 31979244 10.3390/cells9020276 PMC7072450

[4] Ashrafizadeh M, Zarrabi A, Hushmandi K, Hashemi F, Moghadam ER, Owrang M, Hashemi F, Makvandi P, Goharrizi MASB, Najafi M, et al. Lung cancer cells and their sensitivity/resistance to cisplatin chemotherapy: role of microRNAs and upstream mediators. Cell Signal. 2021;78: 109871. 33279671 10.1016/j.cellsig.2020.109871

[5] Paskeh MDA, Mirzaei S, Orouei S, Zabolian A, Saleki H, Azami N, Hushmandi K, Baradaran B, Hashmi M, Aref AR, et al. Revealing the role of miRNA-489 as a new onco-suppressor factor in different cancers based on pre-clinical and clinical evidence. Int J Biol Macromol. 2021;191:727–37. 34562537 10.1016/j.ijbiomac.2021.09.089

[6] Yu M, Du H, Zhang C, Shi Y. miR-192 family in breast cancer: regulatory mechanisms and diagnostic value. Biomed Pharmacother. 2024;175: 116620. 38653113 10.1016/j.biopha.2024.116620

[7] Bartel DP. MicroRNAs: genomics, biogenesis, mechanism, and function. Cell. 2004;116(2):281–97. 14744438 10.1016/s0092-8674(04)00045-5

[8] Achkar NP, Cambiagno DA, Manavella PA. miRNA biogenesis: a dynamic pathway. Trends Plant Sci. 2016;21(12):1034–44. 27793495 10.1016/j.tplants.2016.09.003

[9] Winter J, Jung S, Keller S, Gregory RI, Diederichs S. Many roads to maturity: microRNA biogenesis pathways and their regulation. Nat Cell Biol. 2009;11(3):228–34. 19255566 10.1038/ncb0309-228

[10] Fabian MR, Sonenberg N, Filipowicz W. Regulation of mRNA translation and stability by microRNAs. Annu Rev Biochem. 2010;79:351–79. 20533884 10.1146/annurev-biochem-060308-103103

[11] Yi R, Qin Y, Macara IG, Cullen BR. Exportin-5 mediates the nuclear export of pre-microRNAs and short hairpin RNAs. Genes Dev. 2003;17(24):3011–6. 14681208 10.1101/gad.1158803 PMC305252

[12] Tang C, Yuan P, Wang J, Zhang Y, Chang X, Jin D, Lei P, Lu Z, Chen B. MiR-192–5p regulates the proliferation and apoptosis of cholangiocarcinoma cells by activating MEK/ERK pathway. 3 Biotech. 2021;11(2):99. 33552829 10.1007/s13205-021-02650-w PMC7843823

[13] Loosen SH, Lurje G, Wiltberger G, Vucur M, Koch A, Kather JN, Paffenholz P, Tacke F, Ulmer FT, Trautwein C, et al. Serum levels of miR-29, miR-122, miR-155 and miR-192 are elevated in patients with cholangiocarcinoma. PLoS ONE. 2019;14(1): e0210944. 30653586 10.1371/journal.pone.0210944 PMC6336320

[14] Silakit R, Loilome W, Yongvanit P, Thongchot S, Sithithaworn P, Boonmars T, Koonmee S, Titapun A, Khuntikeo N, Chamadol N, et al. Urinary microRNA-192 and microRNA-21 as potential indicators for liver fluke-associated cholangiocarcinoma risk group. Parasitol Int. 2017;66(4):479–85. 26456596 10.1016/j.parint.2015.10.001

[15] Silakit R, Loilome W, Yongvanit P, Chusorn P, Techasen A, Boonmars T, Khuntikeo N, Chamadol N, Pairojkul C, Namwat N. Circulating miR-192 in liver fluke-associated cholangiocarcinoma patients: a prospective prognostic indicator. J Hepatobiliary Pancreat Sci. 2014;21(12):864–72. 25131257 10.1002/jhbp.145

[16] Xu J, Zou J, Wu L, Lu W. Transcriptome analysis uncovers the diagnostic value of miR-192-5p/HNF1A-AS1/VIL1 panel in cervical adenocarcinoma. Sci Rep. 2020;10(1):16584. 33024199 10.1038/s41598-020-73523-0 PMC7538942

[17] Zhu G, Cao B, Liang X, Li L, Hao Y, Meng W, He C, Wang L, Li L. Small extracellular vesicles containing miR-192/215 mediate hypoxia-induced cancer-associated fibroblast development in head and neck squamous cell carcinoma. Cancer Lett. 2021;506:11–22. 33639203 10.1016/j.canlet.2021.01.006

[18] Huang Q, Hou S, Zhu X, Liu S. MicroRNA-192 promotes the development of nasopharyngeal carcinoma through targeting RB1 and activating PI3K/AKT pathway. World J Surg Oncol. 2020;18(1):29. 32013999 10.1186/s12957-020-1798-y PMC6998165

[19] Chen P, Feng Y, Zhang H, Shi X, Li B, Ju W, Yu X, Zhang N, Luo X. MicroRNA-192 inhibits cell proliferation and induces apoptosis in human breast cancer by targeting caveolin 1. Oncol Rep. 2019;42(5):1667–76. 31485620 10.3892/or.2019.7298 PMC6775803

[20] Hu F, Meng X, Tong Q, Liang L, Xiang R, Zhu T, Yang S. BMP-6 inhibits cell proliferation by targeting microRNA-192 in breast cancer. Biochim Biophys Acta. 2013;1832(12):2379–90. 24012720 10.1016/j.bbadis.2013.08.011

[21] Zhang Y, He Y, Lu LL, Zhou ZY, Wan NB, Li GP, He X, Deng HW. miRNA-192-5p impacts the sensitivity of breast cancer cells to doxorubicin via targeting peptidylprolyl isomerase A. Kaohsiung J Med Sci. 2019;35(1):17–23. 30844143 10.1002/kjm2.12004 PMC11900784

[22] Yang J, Hou G, Chen H, Chen W, Ge J. Circ_0000189 promotes the malignancy of glioma cells via regulating miR-192-5p-ZEB2 Axis. Oxid Med Cell Longev. 2022;2022:2521951. 36193069 10.1155/2022/2521951 PMC9526621

[23] Wang H, Hu Q, Tong Y, Li S, Chen M, Wang B, Li H. LncRNA SOX2-OT regulates miR-192-5p/RAB2A axis and ERK pathway to promote glioblastoma cell growth. Cell Cycle. 2021;20(19):2010–20. 34470582 10.1080/15384101.2021.1965722 PMC8565829

[24] Yang SY, Choi SA, Lee JY, Park AK, Wang KC, Phi JH, Koh EJ, Park WY, Park SH, Hwang DW, et al. miR-192 suppresses leptomeningeal dissemination of medulloblastoma by modulating cell proliferation and anchoring through the regulation of DHFR, integrins, and CD47. Oncotarget. 2015;6(41):43712–30. 26506238 10.18632/oncotarget.6227 PMC4791261

[25] Huang YL, Li XH, Ma H, Yue HY, Hu XY. Metabolites of intestinal microflora upregulate miR-192-5p to suppress proliferation of colon cancer cells via RhoA-ROCK-LIMK2 pathway. Eur Rev Med Pharmacol Sci. 2020;24(4):1794–806. 32141548 10.26355/eurrev_202002_20357

[26] Zheng XF, Liu KX, Wang XM, Zhang R, Li X. MicroRNA-192 acts as a tumor suppressor in colon cancer and simvastatin activates miR-192 to inhibit cancer cell growth. Mol Med Rep. 2019;19(3):1753–60. 30628692 10.3892/mmr.2019.9808

[27] Li P, Ou Q, Braciak TA, Chen G, Oduncu FS. MicroRNA-192-5p is a predictive biomarker of survival for Stage IIIB colon cancer patients. Jpn J Clin Oncol. 2018;48(7):619–24. 29529220 10.1093/jjco/hyy019

[28] Zhao J, Xu J, Zhang R. SRPX2 regulates colon cancer cell metabolism by miR-192/215 via PI3K-Akt. Am J Transl Res. 2018;10(2):483–90. 29511442 PMC5835813

[29] Geng L, Chaudhuri A, Talmon G, Wisecarver JL, Are C, Brattain M, Wang J. MicroRNA-192 suppresses liver metastasis of colon cancer. Oncogene. 2014;33(46):5332–40. 24213572 10.1038/onc.2013.478 PMC4016997

[30] Zhao K, Ye Z, Li Y, Li C, Yang X, Chen Q, Xing C. LncRNA FTX contributes to the progression of colorectal cancer through regulating miR-192-5p/EIF5A2 Axis. Onco Targets Ther. 2020;13:2677–88. 32280242 10.2147/OTT.S241011 PMC7127817

[31] Chiang Y, Song Y, Wang Z, Liu Z, Gao P, Liang J, Zhu J, Xing C, Xu H. microRNA-192, -194 and -215 are frequently downregulated in colorectal cancer. Exp Ther Med. 2012;3(3):560–6. 22969930 10.3892/etm.2011.436 PMC3438543

[32] Ji D, Jiang L, Li Y. MiR-192-5p suppresses the growth of bladder cancer cells via targeting Yin Yang 1. Hum Cell. 2018;31(3):210–9. 29536411 10.1007/s13577-018-0201-6

[33] Fu S, Ma C, Tang X, Ma X, Jing G, Zhao N, Ran J. MiR-192-5p inhibits proliferation, migration, and invasion in papillary thyroid carcinoma cells by regulation of SH3RF3. 2021. Biosci Reports. 10.1042/BSR20210342. 10.1042/BSR20210342 PMC8463656 34486645

[34] Li H, He L, Tuo Y, Huang Y, Qian B. Circular RNA hsa_circ_0000282 contributes to osteosarcoma cell proliferation by regulating miR-192/XIAP axis. BMC Cancer. 2020;20(1):1026. 33097010 10.1186/s12885-020-07515-8 PMC7583201

[35] Shang G, Mi Y, Mei Y, Wang G, Wang Y, Li X, Wang Y, Li Y, Zhao G. MicroRNA-192 inhibits the proliferation, migration and invasion of osteosarcoma cells and promotes apoptosis by targeting matrix metalloproteinase-11. Oncol Lett. 2018;15(5):7265–72. 29731885 10.3892/ol.2018.8239 PMC5920878

[36] Zhou S, Xiong M, Dai G, Yu L, Zhang Z, Chen J, Guo W. MicroRNA-192-5p suppresses the initiation and progression of osteosarcoma by targeting USP1. Oncol Lett. 2018;15(5):6947–56. 29731868 10.3892/ol.2018.8180 PMC5920969

[37] Wang Y, Zhang S, Xu Y, Zhang Y, Guan H, Li X, Li Y, Wang Y. Upregulation of miR-192 inhibits cell growth and invasion and induces cell apoptosis by targeting TCF7 in human osteosarcoma the journal of the international society for oncodevelopmental biology and medicine. Tumour Biol. 2016;37(11):15211–20. 27683056 10.1007/s13277-016-5417-z

[38] Wang Y, Jia LS, Yuan W, Wu Z, Wang HB, Xu T, Sun JC, Cheng KF, Shi JG. Low miR-34a and miR-192 are associated with unfavorable prognosis in patients suffering from osteosarcoma. Am J Transl Res. 2015;7(1):111–9. 25755833 PMC4346528

[39] Senanayake U, Das S, Vesely P, Alzoughbi W, Fröhlich LF, Chowdhury P, Leuschner I, Hoefler G, Guertl B. miR-192, miR-194, miR-215, miR-200c and miR-141 are downregulated and their common target ACVR2B is strongly expressed in renal childhood neoplasms. Carcinogenesis. 2012;33(5):1014–21. 22431721 10.1093/carcin/bgs126

[40] Ni J, Tian W, Liang S, Wang H, Ren Y. Promoter methylation-mediated silencing of the MiR-192-5p promotes endometrial cancer progression by targeting ALX1. Int J Med Sci. 2021;18(12):2510–20. 34104082 10.7150/ijms.58954 PMC8176185

[41] Wang Y, Ma H, Li Y, Su R. MiR-192-5p-modified tumor-associated macrophages-derived exosome suppressed endometrial cancer progression through targeting IRAK1/NF-κB signaling. Reprod Sci. 2022;29(2):436–47. 35000146 10.1007/s43032-021-00789-8

[42] Chen X, Su X, Lin M, Fu B, Zhou C, Ling C, Qian Z, Yao Y. Expression of miR-192-5p in colon cancer serum and its relationship with clinicopathologic features. Am J Transl Res. 2021;13(8):9371–6. 34540055 PMC8430106

[43] Tavakolian S, Goudarzi H, Faghihloo E. Evaluating the expression level of miR-9-5p and miR-192-5p in gastrointestinal cancer: introducing novel screening biomarkers for patients. BMC Res Notes. 2020;13(1):226. 32307002 10.1186/s13104-020-05071-9 PMC7168809

[44] Tian C, Zhang L, Li X, Zhang Y, Li J, Chen L. Low miR-192 expression predicts poor prognosis in pediatric acute myeloid leukemia. Cancer Biomark. 2018;22(2):209–15. 29689705 10.3233/CBM-170657 PMC13078435

[45] Ke S, Li RC, Lu J, Meng FK, Feng YK, Fang MH. MicroRNA-192 regulates cell proliferation and cell cycle transition in acute myeloid leukemia via interaction with CCNT2. Int J Hematol. 2017;106(2):258–65. 28409330 10.1007/s12185-017-2232-2

[46] Jiang F, Li C, Han J, Wang L. Diagnostic value of combination of MicroRNA-192 in urinary sediment and B-ultrasound for bladder cancer. Technol Cancer Res Treat. 2020;19:1533033819894573. 32106776 10.1177/1533033819894573 PMC7052445

[47] Fathullahzadeh S, Mirzaei H, Honardoost MA, Sahebkar A, Salehi M. Circulating microRNA-192 as a diagnostic biomarker in human chronic lymphocytic leukemia. Cancer Gene Ther. 2016;23(10):327–32. 27659777 10.1038/cgt.2016.34

[48] Furuke H, Konishi H, Arita T, Kataoka S, Shibamoto J, Takabatake K, Takaki W, Shimizu H, Yamamoto Y, Komatsu S, et al. Plasma microRNA-192–5p can predict the response to neoadjuvant chemotherapy and prognosis in esophageal cancer. Cancer Sci. 2022. 10.1111/cas.15703. 36533956 10.1111/cas.15703 PMC10067423

[49] Li S, Li F, Niu R, Zhang H, Cui A, An W, Wang X. Mir-192 suppresses apoptosis and promotes proliferation in esophageal aquamous cell caicinoma by targeting Bim. Int J Clin Exp Pathol. 2015;8(7):8048–56. 26339371 PMC4555699

[50] Odenthal M, Bollschweiler E, Grimminger PP, Schröder W, Brabender J, Drebber U, Hölscher AH, Metzger R, Vallböhmer D. MicroRNA profiling in locally advanced esophageal cancer indicates a high potential of miR-192 in prediction of multimodality therapy response. Int J Cancer. 2013;133(10):2454–63. 23649428 10.1002/ijc.28253

[51] Agostini A, Brunetti M, Davidson B, Tropé CG, Eriksson AGZ, Heim S, Panagopoulos I, Micci F. The microRNA miR-192/215 family is upregulated in mucinous ovarian carcinomas. Sci Rep. 2018;8(1):11069. 30038317 10.1038/s41598-018-29332-7 PMC6056508

[52] Chen L, Wang K, Li L, Zheng B, Zhang Q, Zhang F, Chen J, Wang S. Plasma exosomal miR-1260a, miR-7977 and miR-192-5p as diagnostic biomarkers in epithelial ovarian cancer. Future Oncol. 2022;18(26):2919–31. 35893704 10.2217/fon-2022-0321

[53] Song J, Lin Z, Liu Q, Huang S, Han L, Fang Y, Zhong P, Dou R, Xiang Z, Zheng J, et al. MiR-192-5p/RB1/NF-κBp65 signaling axis promotes IL-10 secretion during gastric cancer EMT to induce Treg cell differentiation in the tumour microenvironment. Clin Transl Med. 2022;12(8): e992. 35969010 10.1002/ctm2.992 PMC9377151

[54] Zhang X, Peng Y, Yuan Y, Gao Y, Hu F, Wang J, Zhu X, Feng X, Cheng Y, Wei Y, et al. Histone methyltransferase SET8 is regulated by miR-192/215 and induces oncogene-induced senescence via p53-dependent DNA damage in human gastric carcinoma cells. Cell Death Dis. 2020;11(10):937. 33127874 10.1038/s41419-020-03130-4 PMC7599338

[55] Deng S, Zhang X, Qin Y, Chen W, Fan H, Feng X, Wang J, Yan R, Zhao Y, Cheng Y, et al. miRNA-192 and -215 activate Wnt/β-catenin signaling pathway in gastric cancer via APC. J Cell Physiol. 2020;235(9):6218–29. 32091625 10.1002/jcp.29550

[56] Jin Z, Selaru FM, Cheng Y, Kan T, Agarwal R, Mori Y, Olaru AV, Yang J, David S, Hamilton JP, et al. MicroRNA-192 and -215 are upregulated in human gastric cancer in vivo and suppress ALCAM expression in vitro. Oncogene. 2011;30(13):1577–85. 21119604 10.1038/onc.2010.534 PMC4586057

[57] He J, Wu J, Dong S, Xu J, Wang J, Zhou X, Rao Z, Gao W. Exosome-encapsulated miR-31, miR-192, and miR-375 Serve as clinical biomarkers of gastric cancer. Journal of oncology. 2023;2023:7335456. 36844871 10.1155/2023/7335456 PMC9950326

[58] Chen Q, Ge X, Zhang Y, Xia H, Yuan D, Tang Q, Chen L, Pang X, Leng W, Bi F. Plasma miR-122 and miR-192 as potential novel biomarkers for the early detection of distant metastasis of gastric cancer. Oncol Rep. 2014;31(4):1863–70. 24481716 10.3892/or.2014.3004

[59] Chiang Y, Zhou X, Wang Z, Song Y, Liu Z, Zhao F, Zhu J, Xu H. Expression levels of microRNA-192 and -215 in gastric carcinoma. Pathol Oncol Res. 2012;18(3):585–91. 22205577 10.1007/s12253-011-9480-x

[60] Xie X, Huang N, Zhang Y, Wei X, Gao M, Li M, Ning J, Liu W, Zhao Q, Wang H, et al. MiR-192-5p reverses cisplatin resistance by targeting ERCC3 and ERCC4 in SGC7901/DDP cells. J Cancer. 2019;10(4):1039–51. 30854110 10.7150/jca.25814 PMC6400793

[61] Wang J, Yin G, Bian H, Yang J, Zhou P, Yan K, Liu C, Chen P, Zhu J, Li Z, et al. LncRNA XIST upregulates TRIM25 via negatively regulating miR-192 in hepatitis B virus-related hepatocellular carcinoma. Mol Med. 2021;27(1):41. 33858324 10.1186/s10020-021-00278-3 PMC8050905

[62] Lian J, Jing Y, Dong Q, Huan L, Chen D, Bao C, Wang Q, Zhao F, Li J, Yao M, et al. miR-192, a prognostic indicator, targets the SLC39A6/SNAIL pathway to reduce tumor metastasis in human hepatocellular carcinoma. Oncotarget. 2016;7(3):2672–83. 26684241 10.18632/oncotarget.6603 PMC4823063

[63] Wang X, Wu S, Yang Y, Zhao J. LncRNA CARMN affects hepatocellular carcinoma prognosis by regulating the miR-192-5p/LOXL2 axis. Oxid Med Cell Longev. 2022;2022:9277360. 36254230 10.1155/2022/9277360 PMC9569233

[64] Ashour H, Farghaly ME, Khowailed AA, Aboulhoda BE, Rashed LA, Elsebaie MM, Gaber SS. Modulation of miR-192/NF-κB/ TGF-β/ E-cadherin by thymoquinone protects against diethylnitrosamine/carbon tetrachloride hepatotoxicity. Physiol Internat. 2022. 10.1556/2060.2022.00163. 10.1556/2060.2022.00163 36001412

[65] Gu Y, Wei X, Sun Y, Gao H, Zheng X, Wong LL, Jin L, Liu N, Hernandez B, Peplowska K, et al. miR-192-5p silencing by genetic aberrations is a key event in hepatocellular carcinomas with cancer stem cell features. Cancer Res. 2019;79(5):941–53. 30530815 10.1158/0008-5472.CAN-18-1675 PMC6397664

[66] Yin S, Jin W, Qiu Y, Fu L, Wang T, Yu H. Solamargine induces hepatocellular carcinoma cell apoptosis and autophagy via inhibiting LIF/miR-192-5p/CYR61/Akt signaling pathways and eliciting immunostimulatory tumor microenvironment. J Hematol Oncol. 2022;15(1):32. 35313929 10.1186/s13045-022-01248-w PMC8935708

[67] Yan-Chun L, Hong-Mei Y, Zhi-Hong C, Qing H, Yan-Hong Z, Ji-Fang W. MicroRNA-192-5p promote the proliferation and metastasis of hepatocellular carcinoma cell by targeting SEMA3A. Appli Immunohistochem Mol Morphol. 2017;25(4):251–60. 10.1097/PAI.0000000000000296 26580097

[68] Škrha P, Hořínek A, Anděl M, Škrha J. miRNA-192, miRNA-21 and miRNA-200: new pancreatic cancer markers in diabetic patients? Vnitr Lek. 2015;61(4):351–4. 25894267

[69] Flammang I, Reese M, Yang Z, Eble JA, Dhayat SA. Tumor-Suppressive miR-192-5p Has Prognostic Value in Pancreatic Ductal Adenocarcinoma. Cancers (Basel). 2020;12(6):1693. 32630552 10.3390/cancers12061693 PMC7352756

[70] Zhao C, Zhang J, Zhang S, Yu D, Chen Y, Liu Q, Shi M, Ni C, Zhu M. Diagnostic and biological significance of microRNA-192 in pancreatic ductal adenocarcinoma. Oncol Rep. 2013;30(1):276–84. 23612862 10.3892/or.2013.2420

[71] Feng S, Cong S, Zhang X, Bao X, Wang W, Li H, Wang Z, Wang G, Xu J, Du B, et al. MicroRNA-192 targeting retinoblastoma 1 inhibits cell proliferation and induces cell apoptosis in lung cancer cells. Nucleic Acids Res. 2011;39(15):6669–78. 21511813 10.1093/nar/gkr232 PMC3159440

[72] Zou P, Zhu M, Lian C, Wang J, Chen Z, Zhang X, Yang Y, Chen X, Cui X, Liu J, et al. miR-192-5p suppresses the progression of lung cancer bone metastasis by targeting TRIM44. Sci Rep. 2019;9(1):19619. 31873114 10.1038/s41598-019-56018-5 PMC6928221

[73] Wang T, Li W, Li H, Li W. Dysregulation of exosomal miR-192 and miR-194 expression in lung adenocarcinoma patients. Saudi J Biol Sci. 2021;28(3):1561–8. 33732041 10.1016/j.sjbs.2021.01.013 PMC7938118

[74] Cao J, He Y, Liu HQ, Wang SB, Zhao BC, Cheng YS. MicroRNA 192 regulates chemo-resistance of lung adenocarcinoma for gemcitabine and cisplatin combined therapy by targeting Bcl-2. Int J Clin Exp Med. 2015;8(8):12397–403. 26550150 PMC4612835

[75] Jin H, Qiao F, Wang Y, Xu Y, Shang Y. Curcumin inhibits cell proliferation and induces apoptosis of human non-small cell lung cancer cells through the upregulation of miR-192-5p and suppression of PI3K/Akt signaling pathway. Oncol Rep. 2015;34(5):2782–9. 26351877 10.3892/or.2015.4258

[76] Filipska M, Skrzypski M, Czetyrbok K, Stokowy T, Stasiłojć G, Supernat A, Jassem J, Żaczek AJ, Bigda J. MiR-192 and miR-662 enhance chemoresistance and invasiveness of squamous cell lung carcinoma. Lung Cancer. 2018;118:111–8. 29571988 10.1016/j.lungcan.2018.02.002

[77] Li Y, Zu L, Wu H, Zhang F, Fan Y, Pan H, Du X, Guo F, Zhou Q. MiR-192/NKRF axis confers lung cancer cell chemoresistance to cisplatin via the NF-κB pathway. Thoracic cancer. 2022;13(3):430–41. 34953057 10.1111/1759-7714.14278 PMC8807278

[78] Zhang F, Li Y, Wu H, Qi K, You J, Li X, Zu L, Pan Z, Wang Y, Li Y, et al. MiR-192 confers cisplatin resistance by targeting Bim in lung cancer. Chin J Lung Cancer. 2014;17(5):384–90. 10.3779/j.issn.1009-3419.2014.05.04 PMC6000442 24854555

[79] Chen ZJ, Yan YJ, Shen H, Zhou JJ, Yang GH, Liao YX, Zeng JM, Yang T. miR-192 is overexpressed and promotes cell proliferation in prostate cancer. Med Princ Pract. 2019;28(2):124–32. 30544100 10.1159/000496206 PMC6546031

[80] Sun J, Fan Z, Lu S, Yang J, Hao T, Huo Q. MiR-192 suppresses the tumorigenicity of prostate cancer cells by targeting and inhibiting nin one binding protein. Int J Mol Med. 2016;37(2):485–92. 26743688 10.3892/ijmm.2016.2449

[81] Park MN, Park H, Rahman MA, Kim JW, Park SS, Cho Y, Choi J, Son SR, Jang DS, Shim BS, et al. BK002 induces miR-192-5p-mediated apoptosis in castration-resistant prostate cancer cells via modulation of PI3K/CHOP. Front Oncol. 2022;12: 791365. 35321434 10.3389/fonc.2022.791365 PMC8936126

[82] Zhang X, Peng Y, Huang Y, Deng S, Feng X, Hou G, Lin H, Wang J, Yan R, Zhao Y, et al. Inhibition of the miR-192/215-Rab11-FIP2 axis suppresses human gastric cancer progression. Cell Death Dis. 2018;9(7):778. 30006518 10.1038/s41419-018-0785-5 PMC6045576

[83] Liu J, Wen Y, Liu Z, Liu S, Xu P, Xu Y, Deng S, Hu S, Luo R, Jiang J, et al. VPS33B modulates c-Myc/p53/miR-192-3p to target CCNB1 suppressing the growth of non-small cell lung cancer. Mol Ther Nucleic Acids. 2021;23:324–35. 33425490 10.1016/j.omtn.2020.11.010 PMC7779536

[84] Pan Y, Sun Y, Liu Z, Zhang C. miR-192-5p upregulation mediates the suppression of curcumin in human NSCLC cell proliferation, migration and invasion by targeting c-Myc and inactivating the Wnt/β-catenin signaling pathway. Mol Med Rep. 2020;22(2):1594–604. 32626956 10.3892/mmr.2020.11213

[85] Du X, Qi F, Lu S, Li Y, Han W. Nicotine upregulates FGFR3 and RB1 expression and promotes non-small cell lung cancer cell proliferation and epithelial-to-mesenchymal transition via downregulation of miR-99b and miR-192. Biomed Pharmacother. 2018;101:656–62. 29518612 10.1016/j.biopha.2018.02.113

[86] Fei Z, Wang Y, Gu Y, Xie R, Hao Q, Jiang Y. CircKIF5B promotes hepatocellular carcinoma progression by regulating the miR-192 Family/XIAP Axis. Front Oncol. 2022;12: 916246. 35847962 10.3389/fonc.2022.916246 PMC9281474

[87] Gu Y, Ji F, Liu N, Zhao Y, Wei X, Hu S, Jia W, Wang XW, Budhu A, Ji J, et al. Loss of miR-192-5p initiates a hyperglycolysis and stemness positive feedback in hepatocellular carcinoma. J Exp Clin Cancer Res. 2020;39(1):268. 33256802 10.1186/s13046-020-01785-7 PMC7708108

[88] Sun L, Zhou X, Li Y, Chen W, Wu S, Zhang B, Yao J, Xu A. KLF5 regulates epithelial-mesenchymal transition of liver cancer cells in the context of p53 loss through miR-192 targeting of ZEB2. Cell Adh Migr. 2020;14(1):182–94. 32965165 10.1080/19336918.2020.1826216 PMC7553557

[89] Rokavec M, Bouznad N, Hermeking H. Paracrine induction of epithelial-mesenchymal transition between colorectal cancer cells and its suppression by a p53/miR-192/215/NID1 Axis. Cell Mol Gastroenterol Hepatol. 2019;7(4):783–802. 30831320 10.1016/j.jcmgh.2019.02.003 PMC6468198

[90] Song B, Wang Y, Kudo K, Gavin EJ, Xi Y, Ju J. miR-192 Regulates dihydrofolate reductase and cellular proliferation through the p53-microRNA circuit. Clin Cancer Res. 2008;14(24):8080–6. 19088023 10.1158/1078-0432.CCR-08-1422 PMC2653201

[91] Vajen B, Greiwe L, Schäffer V, Eilers M, Huge N, Stalke A, Schlegelberger B, Illig T, Skawran B. MicroRNA-192-5p inhibits migration of triple negative breast cancer cells and directly regulates Rho GTPase activating protein 19. Genes Chromosomes Cancer. 2021;60(11):733–42. 34296808 10.1002/gcc.22982

[92] Dong RF, Zhuang YJ, Wang Y, Zhang ZY, Xu XZ, Mao YR, Yu JJ. Tumor suppressor miR-192-5p targets TRPM7 and inhibits proliferation and invasion in cervical cancer. Kaohsiung J Med Sci. 2021;37(8):699–708. 34042256 10.1002/kjm2.12398 PMC11896357

[93] Zhang T, Wang C, Wang K, Liang Y, Liu T, Feng L, Yang X. RacGAP1 promotes the malignant progression of cervical cancer by regulating AP-1 via miR-192 and p-JNK. Cell Death Dis. 2022;13(7):604. 35831303 10.1038/s41419-022-05036-9 PMC9279451

[94] Bazavar M, Fazli J, Valizadeh A, Ma B, Mohammadi E, Asemi Z, Alemi F, Maleki M, Xing S, Yousefi B. miR-192 enhances sensitivity of methotrexate drug to MG-63 osteosarcoma cancer cells. Pathol Res Pract. 2020;216(11): 153176. 32861171 10.1016/j.prp.2020.153176

[95] Feinberg-Gorenshtein G, Guedj A, Shichrur K, Jeison M, Luria D, Kodman Y, Ash S, Feinmesser M, Edry L, Shomron N, et al. MiR-192 directly binds and regulates Dicer1 expression in neuroblastoma. PLoS ONE. 2013;8(11): e78713. 24223844 10.1371/journal.pone.0078713 PMC3815303

[96] Jin Y, Lu J, Wen J, Shen Y, Wen X. Regulation of growth of human bladder cancer by miR-192 the journal of the international society for oncodevelopmental biology and medicine. Tumour Biol. 2015;36(5):3791–7. 25566965 10.1007/s13277-014-3020-8

[97] Song Y, Du Y, Qin C, Liang H, Yang W, Lin J, Ding M, Han J, Xu T. Gemcitabine-resistant biomarkers in bladder cancer are associated with tumor-immune microenvironment. Front Develop Biol. 2021;9: 809620. 10.3389/fcell.2021.809620 PMC8814447 35127724

[98] Sun Y, Pan J, Mao S, Jin J. IL-17/miR-192/IL-17Rs regulatory feedback loop facilitates multiple myeloma progression. PLoS ONE. 2014;9(12): e114647. 25489847 10.1371/journal.pone.0114647 PMC4260882

[99] Tuerxun N, Wang J, Qin YT, Zhao F, Wang H, Qu JH, Uddin MN, Hao JP. Identification of key genes and miRNA-mRNA regulatory networks associated with bone marrow immune microenvironment regulations in multiple myeloma by integrative bioinformatics analysis. Hematology. 2022;27(1):506–17. 35536760 10.1080/16078454.2022.2068873

[100] Li Q, Luan Q, Zhu H, Zhao Y, Ji J, Wu F, Yan J. Circular RNA circ_0005774 contributes to proliferation and suppresses apoptosis of acute myeloid leukemia cells via circ_0005774/miR-192-5p/ULK1 ceRNA pathway. Biochem Biophys Res Commun. 2021;551:78–85. 33735626 10.1016/j.bbrc.2021.02.058

[101] Wu W, Deng J, Chen C, Ma X, Yu L, Chen L. Circ_0001602 aggravates the progression of acute myeloid leukemia by regulating the miR-192-5p/ZBTB20 axis. Hematology. 2023;28(1):2240133. 37585722 10.1080/16078454.2023.2240133

[102] Zhang H, Zhang C, Feng R, Zhang H, Gao M, Ye L. Investigating the microRNA-mRNA regulatory network in acute myeloid leukemia. Oncol Lett. 2017;14(4):3981–8. 28989535 10.3892/ol.2017.6686 PMC5620483

[103] Khella HW, Bakhet M, Allo G, Jewett MA, Girgis AH, Latif A, Girgis H, Von Both I, Bjarnason GA, Yousef GM. miR-192, miR-194 and miR-215: a convergent microRNA network suppressing tumor progression in renal cell carcinoma. Carcinogenesis. 2013;34(10):2231–9. 23715501 10.1093/carcin/bgt184

[104] Wu SY, Rupaimoole R, Shen F, Pradeep S, Pecot CV, Ivan C, Nagaraja AS, Gharpure KM, Pham E, Hatakeyama H, et al. A miR-192-EGR1-HOXB9 regulatory network controls the angiogenic switch in cancer. Nat Commun. 2016;7:11169. 27041221 10.1038/ncomms11169 PMC4822037

[105] Gao Y, Zhang H, Zhao S, He D, Gu C. Nanofluorescence probes to detect miR-192/Integrin alpha 1 and their correlations with retinoblastoma. J Biomed Nanotechnol. 2021;17(11):2176–85. 34906278 10.1166/jbn.2021.3185

[106] Honar YS, Javaher S, Soleimani M, Zarebkohan A, Farhadihosseinabadi B, Tohidfar M, Abdollahpour-Alitappeh M. Advanced stage, high-grade primary tumor ovarian cancer: a multi-omics dissection and biomarker prediction process. Sci Rep. 2023;13(1):17265. 37828118 10.1038/s41598-023-44246-9 PMC10570268

[107] Wang L, Peng B, Yan Y, Liu G, Yang D, Wang Q, Li Y, Mao Q, Chen Q. The tRF-3024b hijacks miR-192-5p to increase BCL-2-mediated resistance to cytotoxic T lymphocytes in esophageal squamous cell carcinoma. Int Immunopharmacol. 2024;126: 111135. 37977065 10.1016/j.intimp.2023.111135

[108] Khalafizadeh A, Hashemizadegan SD, Shokri F, Bakhshinejad B, Jabbari K, Motavaf M, Babashah S. Competitive endogenous RNA networks: decoding the role of long non-coding RNAs and circular RNAs in colorectal cancer chemoresistance. J Cell Mol Med. 2024;28(7): e18197. 38506091 10.1111/jcmm.18197 PMC10951891

[109] Rahnama S, Bakhshinejad B, Farzam F, Bitaraf A, Ghazimoradi MH, Babashah S. Identification of dysregulated competing endogenous RNA networks in glioblastoma: a way toward improved therapeutic opportunities. Life Sci. 2021;277: 119488. 33862117 10.1016/j.lfs.2021.119488

[110] Ye M, Zhang J, Zhang J, Miao Q, Yao L, Zhang J. Curcumin promotes apoptosis by activating the p53-miR-192-5p/215-XIAP pathway in non-small cell lung cancer. Cancer Lett. 2015;357(1):196–205. 25444916 10.1016/j.canlet.2014.11.028

[111] Guo Q, Jin Y, Chen X, Ye X, Shen X, Lin M, Zeng C, Zhou T, Zhang J. NF-κB in biology and targeted therapy: new insights and translational implications. Signal Transduct Target Ther. 2024;9(1):53. 38433280 10.1038/s41392-024-01757-9 PMC10910037

[112] Yang Q, Zhuge X, Lin W, Yu W, Zhu Y, Shi C, Shi Z. Hydrogel-based miR-192 delivery inhibits the development of hepatocellular carcinoma by suppressing the GSK3β/Wnt/β-catenin pathway. Neoplasma. 2023;70(4):555–65. 37789778 10.4149/neo_2023_230317N150

[113] Sasaki CT, Vageli DP. miR-21, miR-155, miR-192, and miR-375 deregulations related to NF-kappaB activation in gastroduodenal fluid-induced early preneoplastic lesions of laryngeal mucosa in vivo. Neoplasia. 2016;18(6):329–38. 27292022 10.1016/j.neo.2016.04.007 PMC4909705

[114] Doukas SG, Vageli DP, Sasaki CT. NF-κB inhibition reverses acidic bile-induced miR-21, miR-155, miR-192, miR-34a, miR-375 and miR-451a deregulations in human hypopharyngeal cells. J Cell Mol Med. 2018;22(5):2922–34. 29516639 10.1111/jcmm.13591 PMC5908126

[115] Zhang X, Peng Y, Huang Y, Yang M, Yan R, Zhao Y, Cheng Y, Liu X, Deng S, Feng X, et al. SMG-1 inhibition by miR-192/-215 causes epithelial-mesenchymal transition in gastric carcinogenesis via activation of Wnt signaling. Cancer Med. 2018;7(1):146–56. 29239144 10.1002/cam4.1237 PMC5773975

[116] Zhang J, Zhao CY, Zhang SH, Yu DH, Chen Y, Liu QH, Shi M, Ni CR, Zhu MH. Upregulation of miR-194 contributes to tumor growth and progression in pancreatic ductal adenocarcinoma. Oncol Rep. 2014;31(3):1157–64. 24398877 10.3892/or.2013.2960

[117] Kandimalla R, Shimura T, Mallik S, Sonohara F, Tsai S, Evans DB, Kim SC, Baba H, Kodera Y, Von Hoff D, et al. Identification of serum miRNA signature and establishment of a nomogram for risk stratification in patients With pancreatic ductal adenocarcinoma. Ann Surg. 2022;275(1):e229–37. 32398486 10.1097/SLA.0000000000003945 PMC7648727

[118] Khan IA, Rashid S, Singh N, Rashid S, Singh V, Gunjan D, Das P, Dash NR, Pandey RM, Chauhan SS, et al. Panel of serum miRNAs as potential non-invasive biomarkers for pancreatic ductal adenocarcinoma. Sci Rep. 2021;11(1):2824. 33531550 10.1038/s41598-021-82266-5 PMC7854650

[119] Fründt T, Krause L, Hussey E, Steinbach B, Köhler D, von Felden J, Schulze K, Lohse AW, Wege H, Schwarzenbach H. Diagnostic and prognostic value of miR-16, miR-146a, miR-192 and miR-221 in exosomes of hepatocellular carcinoma and liver cirrhosis patients. Cancers (Basel). 2021;13(10):2484. 34069692 10.3390/cancers13102484 PMC8161187

[120] Li G, Yin J, Wu Z, Li S, He A, Sun Z. Expression level of miRNA in the peripheral blood of patients with multiple myeloma and its clinical significance. Am J Transl Res. 2021;13(5):5343–9. 34150128 PMC8205780

[121] Wang G, Chan ES, Kwan BC, Li PK, Yip SK, Szeto CC, Ng CF. Expression of microRNAs in the urine of patients with bladder cancer. Clin Genitourin Cancer. 2012;10(2):106–13. 22386240 10.1016/j.clgc.2012.01.001

[122] Poel D, Rustenburg F, Sie D, van Essen HF, Eijk PP, Bloemena E, Elhorst Benites T, van den Berg MC, Vergeer MR, Leemans RC, et al. Expression of let-7i and miR-192 is associated with resistance to cisplatin-based chemoradiotherapy in patients with larynx and hypopharynx cancer. Oral Oncol. 2020;109: 104851. 32585557 10.1016/j.oraloncology.2020.104851

